# Abiotic factors shape mosquito microbiomes that enhance host development

**DOI:** 10.1093/ismejo/wrae181

**Published:** 2024-09-24

**Authors:** Nicola G Kriefall, Priscilla S Seabourn, Nicole M Yoneishi, Kahiwahiwa Davis, Kirsten K Nakayama, Danya E Weber, Nicole A Hynson, Matthew C I Medeiros

**Affiliations:** Pacific Biosciences Research Center, University of Hawai‘i at Mānoa, 1993 East-West Road, Honolulu, HI 96822, United States; Pacific Biosciences Research Center, University of Hawai‘i at Mānoa, 1993 East-West Road, Honolulu, HI 96822, United States; Pacific Biosciences Research Center, University of Hawai‘i at Mānoa, 1993 East-West Road, Honolulu, HI 96822, United States; Center for Microbiome Analysis through Island Knowledge and Investigation, University of Hawai‘i at Mānoa, 1993 East-West Road, Honolulu, HI 96822, United States; Pacific Biosciences Research Center, University of Hawai‘i at Mānoa, 1993 East-West Road, Honolulu, HI 96822, United States; Pacific Biosciences Research Center, University of Hawai‘i at Mānoa, 1993 East-West Road, Honolulu, HI 96822, United States; Center for Microbiome Analysis through Island Knowledge and Investigation, University of Hawai‘i at Mānoa, 1993 East-West Road, Honolulu, HI 96822, United States; Pacific Biosciences Research Center, University of Hawai‘i at Mānoa, 1993 East-West Road, Honolulu, HI 96822, United States; Pacific Biosciences Research Center, University of Hawai‘i at Mānoa, 1993 East-West Road, Honolulu, HI 96822, United States; Center for Microbiome Analysis through Island Knowledge and Investigation, University of Hawai‘i at Mānoa, 1993 East-West Road, Honolulu, HI 96822, United States; Pacific Biosciences Research Center, University of Hawai‘i at Mānoa, 1993 East-West Road, Honolulu, HI 96822, United States; Center for Microbiome Analysis through Island Knowledge and Investigation, University of Hawai‘i at Mānoa, 1993 East-West Road, Honolulu, HI 96822, United States

**Keywords:** microbiome assembly, environment, Aedes albopictus, developmental success

## Abstract

Metazoans rely on interactions with microorganisms through multiple life stages. For example, developmental trajectories of mosquitoes can vary depending on the microorganisms available during their aquatic larval phase. However, the role that the local environment plays in shaping such host-microbe dynamics and the consequences for the host organism remain inadequately understood. Here, we examine the influence of abiotic factors, locally available bacteria, and their interactions on the development and associated microbiota of the mosquito *Aedes albopictus*. Our findings reveal that leaf detritus infused into the larval habitat water, sourced from native Hawaiian tree ‘ōhi‘a lehua *Metrosideros polymorpha*, invasive strawberry guava *Psidium cattleianum*, or a pure water control, displayed a more substantial influence than either temperature variations or simulated microbial dispersal regimes on bacterial community composition in adult mosquitoes. However, specific bacteria exhibited divergent patterns within mosquitoes across detrital infusions that did not align with their abundance in the larval habitat. Specifically, we observed a higher relative abundance of a *Chryseobacterium* sp. strain in mosquitoes from the strawberry guava infusion than the pure water control, whereas the opposite trend was observed for a *Pseudomonas* sp. strain. In a follow-up experiment, we manipulated the presence of these two bacterial strains and found larval developmental success was enhanced by including the *Chryseobacterium* sp. strain in the strawberry guava infusion and the *Pseudomonas* sp. strain in the pure water control. Collectively, these data suggest that interactions between abiotic factors and microbes of the larval environment can help shape mosquito populations' success.

## Introduction

The biological functioning of many metazoans is tied to microbial symbionts [1]. However, the nature of these relationships can change depending on environmental context, for instance by rendering certain host-microorganism relationships more beneficial than others [1–3]. Given the complex nature of these associations and their relevance for metazoans, it is crucial to deepen our understanding of the ecological drivers of host-associated microbiome diversity and fitness outcomes.

Closer examination of the mosquito-associated microbiome is particularly advantageous as manipulating microbes can disrupt this host’s ability to vector human pathogens [4–6]. Accordingly, prior work indicates that the bacterial microbiome has wide-ranging impacts on this holometabolous insect, affecting critical areas such as development, adult digestion, lifespan, and reproductive output [7–9]. The majority of hosted bacterial diversity in early life stages appears sourced from locally available microbes [10] and can vary with environmental gradients more strongly than by host species [11, 12], but typically only a subset of this diversity persists into adulthood [7, 13]. The specific factors driving the maintenance of bacterial community turnover and the associated consequences for mosquito fitness remain inadequately described.

Axenic or gnotobiotic mosquito larvae provide tractable study systems to better understand environmental pressures in shaping mosquito-microbe interactions and further elucidate the specific roles of microbial taxa [14]. Recent work has demonstrated that axenic mosquito larvae can fail to develop, but development can be rescued by rearing without light to prevent degradation of riboflavin or by providing food in a semi-solid form [7, 14–16]. In addition, a variety of bacteria and microeukaryotes can rescue development, but not all, and there can be tradeoffs in relative developmental success [17–19]. Concurrently, the developmental schedule of xenic mosquito larvae is generally linked to abiotic variables, including temperature [20], resource availability [21], and chemistry of the aquatic environment [22]. This body of work displays the tight links between mosquito development and various abiotic and biotic conditions but leaves open the question of whether the relative benefits of microbes for development may change with environmental context.

The objective of our study is to more closely define the relative, and putatively interacting, impacts of larval habitat factors on adult mosquito emergence success and microbiome assembly. We use the tiger mosquito *Ae. albopictus*, which holds importance worldwide as a vector of dengue and chikungunya viruses [23, 24] and locally in the Hawaiian Islands where it is invasive. We rear gnotobiotic larvae under full cross-factorial treatments that manipulate environmental variables and the composition of bacteria available in the water column. Specifically, we alter ambient temperature and infuse water with botanical detritus from native or invasive sources, which are important variables amid shifting climates and landscapes [20–22]. In addition, we simulate microbial dispersal by re-allocating homogenized water across treatments, with or without filtration, providing extirpated microorganisms an opportunity to reestablish. In a follow-up experiment, we test whether the inverse relative abundance of two strains (*Pseudomonas* sp., *Chryseobacterium* sp.) observed in adult mosquitoes from contrasting detrital environments could be explained by improved host development due to interactions with these microbes. Our results indicate that distinct combinations of bacteria with abiotic habitat factors underscore variation in host development.

## Materials and methods

### Experiment I: mosquito microbiome assembly

Approximately 3000 eggs were collected from the F8 generation of an *Ae. albopictus* colony, which was originally isolated from a wild population in Mānoa, Hawai‘i and was superinfected with *Wolbachia* strains wAlbA and wAlbB. Eggs were sterilized by submergence in 70% ethanol for 5 min, 3% bleach for 3 min, 0.1% D-256 (Vedco, USA) for 3 min, and sterile phosphate-buffered saline for three washes of 2 min each. Eggs hatched for 72 h in sterile T-250 flasks containing sterile water and autoclave-sterilized larval food (0.83 mg/ml of 1:1 brewer’s yeast:bovine liver powder) in Percival I-41VL environmental chambers with a humidity of >60% and the following daily cycle: 12 h light at 27°C:12 h dark at 23.5°C. Sterility, aside from intracellular *Wolbachia*, was confirmed by documenting the arrested development of first-instar larvae [7, 19].

Ten larvae were allocated to each experimental mesocosm, which consisted of 60 ml of aquatic media in a sterile T-250 flask. Each mesocosm was exposed to one of 12 full factorial treatments (three detrital infusions, two temperature regimes, and two dispersal levels; see below), with six replicates each (72 mesocosms total). Larval food (described above) was provided at a rate of 1.2 mg/larva every 4 days.

Botanical detritus infusions were composed of ‘ōhi‘a lehua (OL; *Metrosideros polymorpha*) or strawberry guava (SG; *Psidium cattleianum*) leaves (see supplementary information). OL, a foundational native Hawaiian tree, and SG, an invasive tree from the tropical Americas, both grow in near-monocultures across mountainous Hawaiian environments, where the detritus of mosquito habitats may be largely composed of the dominant trees' leaves. Autoclaved Millipore-filtered pure water (PW) comprised the third infusion type. Infusions were double-filtered (0.2 μm, then 0.1 μm) to reduce particulates and microorganisms.

Temperature was manipulated within two separate environmental chambers with the same daily settings as above, except with 25°C diurnal/21°C nocturnal temperatures for the cool treatment and 30°C diurnal/26°C nocturnal for the warm treatment. These temperature regimes mirror the upper and lower elevational limits of *Ae. albopictus’* core range in Hawaiʻi.

Every mesocosm received an inoculation of 23 bacterial strains from 13 genera and nine families, all associated with wild *Ae. albopictus* microbiomes in Hawai‘i [10, 25–27], originally isolated from the midguts of female adults on O‘ahu (Tables 1, S1; see supplementary information). Wild Hawaiian *Ae. albopictus* adults can host an average richness of one to 86 ASVs [10, 25, 27] and have been associated with 38 unique genera worldwide [28], but we were limited by the fraction that was amenable to culturing efforts. To create the inoculum, the cell concentration of each culture was assessed using a hemocytometer (Paul Marienfeld, Germany) with Erythrosin B cell stain. The cultures were diluted in LB broth to 10^6^ cells/ml each and pooled. A 60 μl amount of this inoculum (Regional Taxa Pool of Experiment I; RTP-I) was added to the water of each mesocosm for a final working concentration of 10^3^ cells/ml.

**Table 1:** Bacterial cultures pooled for the Experiment I inoculum. Culture information includes their respective genus (or family where genus was not distinguishable; indicated by “^#^”), shorthand label, and mosquito species source (*Ae. albo: Aedes albopictus*, *Ae. vexa: Aedes vexans*, *C. quin: Culex quinquefasciatus*, *W. mitc: Wyeomyia mitchellii*). Cultures that could not be distinguished from each other within the experiment's 16S amplicon sequence variant (ASV) data were collapsed. The “Present in mosq., water” column indicates whether the ASVs were detected in the mosquito and/or mesocosm water samples of Experiment I. For Experiment II, “^” indicates the six cultures pooled and “^*^” indicates the two cultures whose presence was manipulated. Further taxonomic and relative abundance data are available in Table S1.

**Genus/family**	**Culture label**	**Mosquito source**	**Collapsed ASVs**	**Present in mosq., water**
*Asaia*	ASAI1^ ASAI2	*Ae. albo* *Ae. albo*	ASAI1/2	Both
*Asaia*	ASAI3	*Ae. albo*	ASAI3	Both
*Bacillus*	BACI1	*Ae. albo*	BACI1	Water only
*Carnobacterium*	CARN1^	*W. mitc*	CARN1	Both
*Chryseobacterium*	CHRY1^*^	*Ae. albo*	CHRY1	Both
*Enterobacter*	ENTE1 ENTE2^	*Ae. vexa* *Ae. albo*	ENTE1/2	Both
*Halomonadaceae* ^#^	HALO1	*W. mitc*	HALO1	Missing
*Klebsiella*	KLEB1	*Ae. albo*	KLEB1	Both
*Kosakonia*	KOSA1^	*Ae. vexa*	KOSA1	Both
*Kosakonia*	KOSA2	*Ae. albo*	KOSA2	Both
*Pantoea*	PANT1 PANT2 PANT3	*C. quin* *C. quin* *Ae. albo*	PANT1/2/3	Both
*Pantoea*	PANT4	*Ae. albo*	PANT4	Water only
*Proteus*	PROT1	*W. mitc*	PROT1	Water only
*Pseudomonas*	PSEU1 PSEU3	*C. quin* *Ae. albo*	PSEU1/3	Water only
*Pseudomonas*	PSEU2^*^	*W. mitc*	PSEU2	Both
*Stenotrophomonas*	STEN1^	*Ae. albo*	STEN1	Both
*Stenotrophomonas*	STEN2	*Ae. albo*	STEN2	Both
*Xanthomonadaceae* ^#^	XANT1	*Ae. vexa*	XANT1	Both

For microbial “dispersal” levels, 100 μl of aquatic media from each mesocosm was pooled every 2 days for 20 experimental days. Half of the mixture was double-filtered (as above) and 70 μl was distributed into each low dispersal mesocosm. 70 μl of the unfiltered media was distributed into each high dispersal mesocosm.

### Experiment I sample collection & sequencing

Pupae were collected within 12–24 h of pupation, rinsed twice in sterile water, and allowed to emerge separately in 30 ml of sterile water. Adult mosquitoes (*n* = 197) were collected within 12–24 h of emergence and stored at −80°C until processing. Other samples collected were the RTP-I inoculum (*n* = 1), infusion water replicates (*n* = 3/infusion; *n* = 9 total), hatching flask water (*n* = 5), larval food water on days 0, 10, and 20 (*n* = 3), and water from each of the 72 mesocosms on days 4, 12, and 20 (*n* = 216). DNA was extracted from all samples using the NucleoMag Tissue kit (Macherey-Nagel, Germany) on a KingFisher Flex (Thermo Fisher Scientific, USA). 16S rRNA gene libraries (V4 region 515F-806R; [29, 30]) were prepared from these samples, as well as five negative controls (three from extraction, two from PCR) and one positive control (Zymo Research, USA). The resulting library was checked for quality and quantity using a Bioanalyzer High Sensitivity Kit (Agilent Technologies, USA) and sequenced by the Advanced Studies in Genomics, Proteomics, and Bioinformatics core facility at the University of Hawai‘i at Mānoa using a MiSeq (PE300 v3; Illumina, USA). Total *Wolbachia* sp. load was quantified using qPCR with *Wolbachia*-specific primers *W*-Spec-16S-F and *W*-Spec-16S-R [31]. Primers of the actin gene, alb-act-F and alb-act-R [32], were used to quantify host genomic copies. See supplementary information for more details.

### Experiment I bioinformatics

16S rRNA gene reads were processed by the bioinformatics pipeline MetaFlow|mics [33]. Annotated R [34] code using the amplicon sequence variants (ASVs) output from this pipeline is available at github.com/nicfall/mosquito_microbes. Taxonomy was assigned using the SILVA database v138.1 [35]. Post-quality control (see supplementary information), 69 ASVs remained across 195 mosquito samples and 49 ASVs remained across 211 mesocosm water samples. We intentionally introduced 23 bacteria, but the mosquitoes endogenously hosted *Wolbachia* and additional taxa may have been sourced from previously undetected strain diversity within the bacterial cultures, infusions post-filtration, or technical contamination. We used *phylogeny.fr* [36] to align ASVs in the RTP-I sample with previous Sanger sequencing data from the 16S rRNA genes of the individual cultures that composed the RTP-I (Table S1). These alignments, in tandem with samples from the infusions, hatching flask, and food water, were used to classify ASVs as intentionally introduced or extraneous.

To examine α-diversity differences, we used *phyloseq* [37] to calculate ASV richness and Simpson’s index. Both α-diversity analyses were repeated on samples rarefied to 9200 reads to ensure that results were independent of sequencing depth. To evaluate differences in community composition using taxa with higher representation for statistical robustness, ASVs present in at least 3.5% of samples (i.e. >6 for mosquitoes and >7 for mesocosm water) were retained, which included 29 ASVs for mosquitoes and 39 for water samples.

### Experiment I statistical analyses

To compare rates of emergence (days), we used a mixed-effects Cox model from package *coxme* [38]. Emergence success was assessed with a generalized linear mixed model (GLMM) assuming a binomial error distribution (logit link function) within package *glmmTMB* [39]. For comparing *Wolbachia* sp. abundance (qPCR results) and α-diversity metrics (ASV richness and Simpson’s index) across treatments, we used linear mixed models within *glmmTMB* [39]. All models included fixed effects of infusion, temperature, dispersal, time (where applicable), and host sex (where applicable), with mesocosm ID as a random effect. Pairwise interaction terms between fixed effects were included if they improved model fit (indicated by lower AIC). For the non-rarefied dataset, analysis of ASV richness included a term that offset results by the natural logarithm of the total counts of each sample. Simpson’s index analysis was conducted on transformed data (Ordered Quantile normalization; [40]) as it improved the distribution of residuals to meet mixed model assumptions.

To assess compositional differences in mosquito-associated and mesocosm water bacterial communities between groups (i.e. sex (where applicable), time, dispersal, temperature, and infusion), we applied a GLMM framework with a binomial error distribution and logit link function to the >3.5% prevalence dataset using *glmmTMB* [39]. In the model, the dependent variable was the proportion of read counts from a specific bacterial taxon in a specific sample (successes were read counts of a particular ASV and failures were counts from all other ASVs). We included a series of random interactions between taxa and each treatment of interest. Statistical inference was performed by comparing nested models with and without a focal random interaction, using log-likelihood ratio tests assuming a χ^2^-distribution. Additional random effects of mesocosm, interactions between taxa and mesocosm, taxon ID, and row ID (to model overdispersion) were included. See [41–43] for more information on mixed model applications to microbiomes. GLMM results were used to calculate adjusted predicted probabilities of encountering each ASV across treatments with the *ggeffects* package [44]. A second GLMM included the random effects of bacterial clade (groups of 3–5 closely related taxa) and interactions between clade and each treatment to assess variance attributed to phylogenetic patterns. Lastly, to compare results to other community dissimilarity metrics, Aitchison and weighted UniFrac distances were calculated and compared between groups (infusion, temperature, dispersal), where data were aggregated by mesocosm to account for lack of independence of larvae from the same mesocosm. Aitchison distances were also compared between the >3.5% prevalence dataset and the full dataset to ensure filtering low-prevalence taxa did not introduce biases.

### Experiment II: mosquito development

Larval mesocosms consisted of two aquatic infusions (SG or PW) and four bacterial inocula (see below) in a fully factorial design. Each of these eight treatment combinations was replicated six times (48 mesocosms total). Mesocosms were prepared and maintained in the same manner as in Experiment I, with the following exceptions. The base Regional Taxa Pool of Experiment II (RTP-II) consisted of KOSA1, STEN1, ENTE2, CARN1, and ASAI1. This pool represented taxa that commonly colonized mosquitoes in Experiment I in SG and PW conditions. The four inocula consisted of one of the following: RTP-II, RTP-II + CHRY1, RTP-II + PSEU2, or RTP-II + BOTH, where “BOTH” included both CHRY1 and PSEU2. Mesocosms were maintained at a 12:12 h 27.5°C diurnal/23.5°C nocturnal cycle.

For each infusion type (SG or PW), we used a GLMM [39] to evaluate whether CHRY1 and PSEU2 treatments altered the probability that larvae would develop into adults. The GLMM assumed a binomial error distribution with a logit link function, included mesocosm ID as a random effect, and the interaction between CHRY1 and PSEU2 was included if it improved model fit (assessed by AIC). Next, to evaluate whether CHRY1 and PSEU2 treatments affected the rate of development within each infusion, we used a mixed-effects Cox model [38] with the same random and fixed effects as in the GLMM.

## Results

### *Ae. albopictus* emergence varies across infusion and temperature treatments

**Figure 1:**
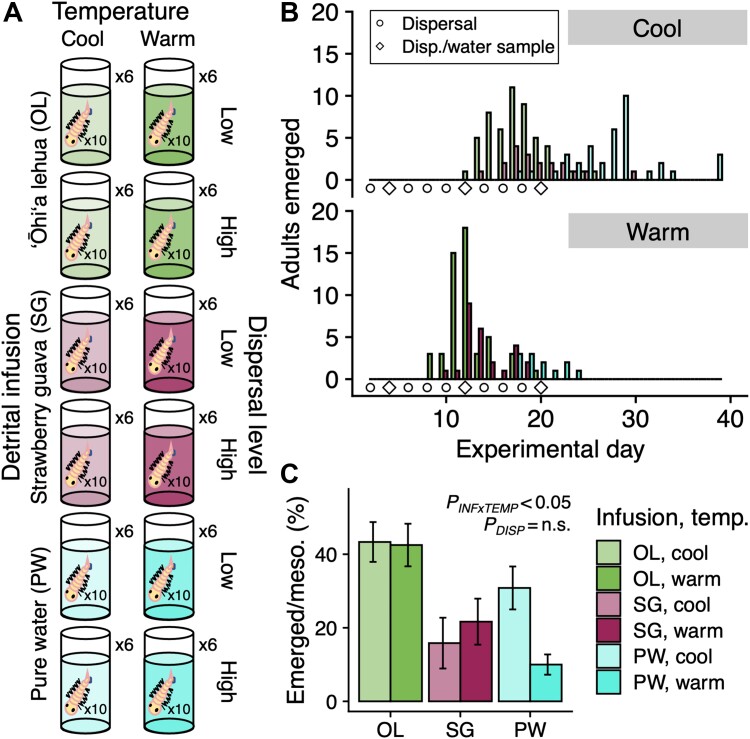
**Overview of experiment I: Mosquito microbiome assembly.** (**A**) Mesocosms with each of 12 fully factorial treatments: Three detrital infusions (OL: ‘ōhi‘a lehua, SG: strawberry guava, PW: pure water), two temperatures regimes (cool: 21–25°C, warm: 26–30°C), and two microbial dispersal levels (low: filtration during dispersal, high: no filtration). Each mesocosm had six replicates and contained 10 *Ae. albopictus* larvae (72 mesocosms; 720 larvae total). (**B**) number of adult mosquitoes that emerged per experimental day, colored by infusion and split by cool (top) and warm (bottom) treatments. On the timeline, dispersal events between mesocosms are indicated every 2 days (circle & diamond shapes) in conjunction with collections of mesocosm water samples for sequencing on days 4, 12, and 20 (diamond shape). (**C**) bars indicate the average percentage of larvae that emerged as adults per mesocosm, with error bars displaying the standard error. The right legend in **C** applies to all panels.

Mosquito emergence success was low across Experiment I, with 197 adults emerging from 720 larvae placed into mesocosms (27.4%). Success of emergence did not vary significantly with dispersal treatments (*P* = 0.53), but there was a significant interaction between infusion and temperature (*P* < 0.05) (Fig. 1). This interaction was driven by a reduction in the emergence success of cool SG mesocosms (15.8 ± 23.9%; mean ± s.d.) relative to warm SG mesocosms (21.7 ± 21.7%), and warm PW mesocosms (10.0 ± 9.5%) relative to cool PW mesocosms (30.8 ± 20.2%) (Fig. 1). The difference in emergence success between cool and warm OL mesocosms was negligible (43.3 ± 18.7% vs. 42.5 ± 20.0%, respectively). OL mesocosms had higher emergence success across both temperatures when compared to SG and PW mesocosms (Fig. 1). Days taken for mosquito adults to emerge varied significantly by infusion and temperature treatments (both *P* < 0.001) (Figs. 1, S1), but not by dispersal (*P* = 0.06). Emergence times were quickest in the OL treatment (15.1 ± 3.7 d), followed by SG (16.7 ± 4.1 d), and PW (25.5 ± 5.3 d) (Figs. 1, S1). Within each infusion type, warm mesocosms prompted faster emergence times than cool mesocosms by a range of 5.3 to 7.5 d (Figs. 1, S1).

### Inoculated bacteria in mosquito & environmental microbiomes

All 23 individual culture sequences aligned phylogenetically (> 99.6% percent identity) with at least one ASV in the RTP-I sample (Tables 1, S1, Fig. S2). Multiple ASVs aligning to culture sequences, likely due to base pair differences outside the 515–806 V4 region, collapsed possible RTP-I taxa from 23 to 18 (Tables 1, S1, Fig. S2). Across the mosquito and mesocosm water samples, the 18 ASVs from the RTP-I sample comprised 96.4% of non-*Wolbachia* reads (Fig. S3), indicating nearly all diversity was sourced from our inoculation.

### *Ae. albopictus* microbiota in Experiment I

**Figure 2:**
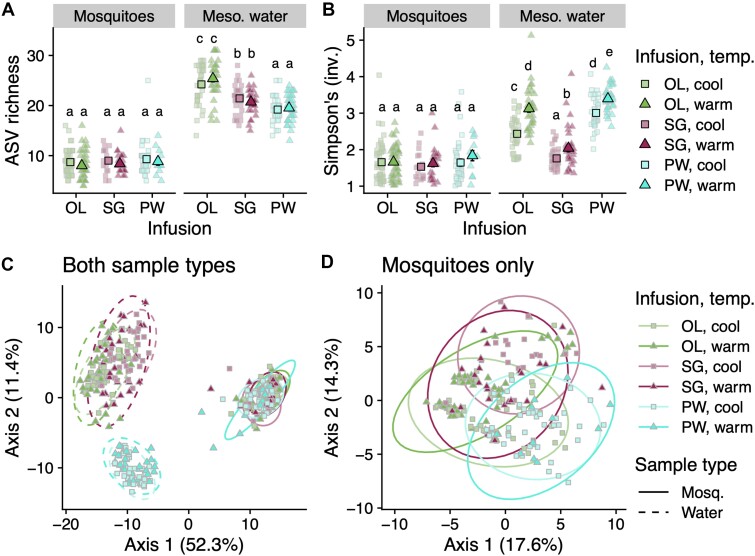
**Bacterial α- and β-diversity across treatments in adult mosquitoes and larval habitat water.** Top panels display α-diversity in terms of **A**) ASV richness and **B**) Simpson’s index (inverted), where central symbols represent the mean and error bars denote standard error. Differences in letters above the symbols indicate a statistically significant Tukey *post hoc* comparison (*P* < 0.05), where tests were run separately for mosquito and mesocosm water (“Meso. Water”) samples. The legend to the right applies to both top panels. Bottom panels display β-diversity between **C**) both sample types and **D**) mosquito samples only, displayed as principal coordinate analysis (PCoA) plots of Aitchison distances between samples. Ellipses indicate 95% confidence intervals. The legend on the right applies to both bottom panels. (OL: ‘ōhi‘a lehua, SG: strawberry guava, PW: pure water.)

In adult mosquitoes, ASV richness did not differ across any treatments (infusion: *P* = 0.78, temperature: *P* = 0.64, dispersal: *P* = 0.24), time (*P* = 0.40), or sex (*P* = 0.53) (Fig. 2). For Simpson’s index (inverted), mosquito microbiomes only differed by sex, where it was higher in females (*P* < 0.001), but did not differ by other factors (infusion: *P* = 0.26, temperature: *P* = 0.58, dispersal: *P* = 0.10, time: *P* = 0.31) (Fig. 2). Relative effect sizes and statistical significance of these comparisons were equivalent when examining rarefied data.

**Figure 3:**
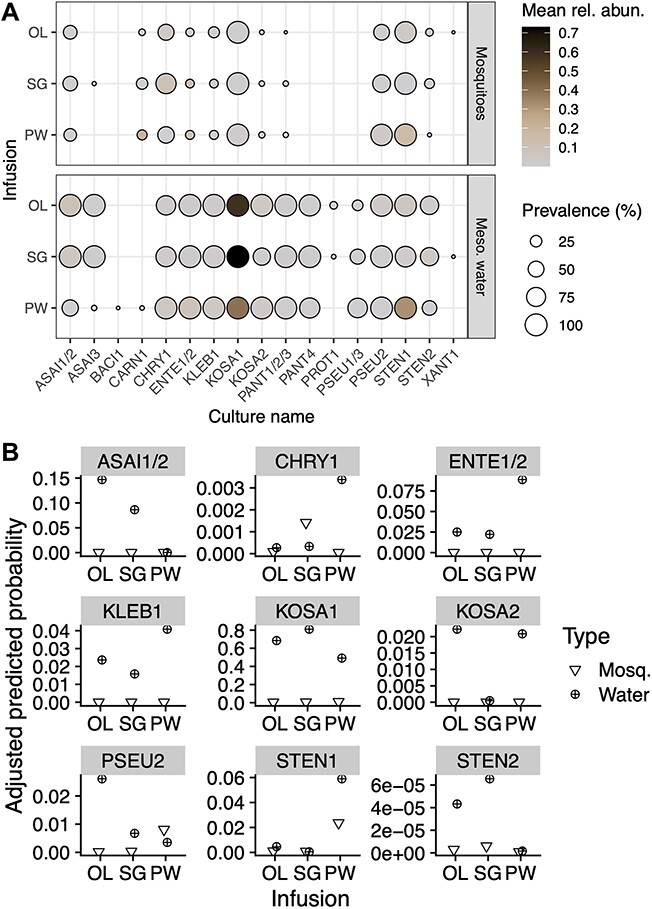
**Inoculated bacterial taxa in mosquitoes and mesocosm water across detrital infusions.** (**A**) inoculated bacteria (RTP-I) within mosquitoes (top) and mesocosm water (bottom) samples, grouped by infusion types (OL: ‘ōhi‘a lehua, SG: strawberry guava, PW: pure water). Circles scale in size with prevalence (% of samples) and are colored by mean relative abundance. (**B**) for each RTP-I taxon present in at least 3.5% of samples, predicted probabilities of encountering a single sequencing read from that taxon per infusion type based on GLMM results.

GLMM analysis revealed that adult mosquito microbiotas varied with the infusion type (*P* < 0.001), sex (*P* < 0.001), and time period of emergence (*P* < 0.05) (Table S2). Temperature was marginally insignificant (*P* = 0.054) and dispersal level was not significantly influential (*P* = 0.36). Of these variables, and in agreement with both weighted UniFrac and Aitchison distances, the greatest variation in the composition of mosquito-associated microbiota was attributed to infusion (Fig. 2; Table S2). Bacterial strains CHRY1 and PSEU2 showed the starkest changes in the mosquito microbiome across infusion treatments, which were not explained by corresponding increases in the mesocosm water (Figs. 3, S4–5). The likelihood of detecting CHRY1 in symbiosis within an SG mesocosm was 4.3 times higher than in the free-living condition in the same mesocosm; for PSEU2, this likelihood was 2.3 times higher within a PW mesocosm (Figs. 3, S5). In contrast, the predicted probabilities of detecting all extraneous, i.e. not intentionally inoculated, ASVs were nearly zero across all infusions (Fig. S6). Relative levels of explained variance and statistical significance for Aitchison distances were equivalent between the full and > 3.5% prevalence datasets across all treatment groups (Fig. S7).

The effect of sex on mosquito microbiome composition was largely driven by the two main *Wolbachia* lineages. WAlbB composed a larger proportion of the adult male *Ae. albopictus* microbiome, whereas wAlbA was more common in the female microbiome (Figs. S4, S8). The temperature of the larval water trended towards affecting the composition of the mosquito microbiome (Fig. S4, Table S2). KLEB1 and PSEU2 were more enriched in mosquitoes from the cool treatment; CARN1 and STEN1 were more enriched in the warmer treatment (Fig. S4). Microbiome variation across experimental groups was not significantly associated with bacterial cladal groupings (infusion: *P* = 0.97, temperature: *P* = 1, time: *P* = 0.08, dispersal: *P* = 1, sex: *P* = 0.65), i.e. individual ASVs exhibited unique responses despite close phylogenetic relationships (Fig. S9). Total *Wolbachia* sp. abundance (based on qPCR) did not vary significantly with treatments, time, or sex (see supplementary information).

### Aquatic habitat microbiota in Experiment I

Mesocosm water alpha diversity results are reported in more detail in the supplementary information (also Figs. 2, S10). GLMM analysis showed that mesocosm water microbiotas varied with infusion type, temperature, time period, and dispersal (all *P* < 0.001; Fig. 2, Table S3). Of the inoculated taxa, STEN1 and CHRY1 were overrepresented in PW mesocosms; ASAI3 and ASAI1/2 were overrepresented in both SG and OL mesocosms, among others (Fig. S4). The taxa most affected by temperature in the water column were PSEU1/3 and KLEB1, which were enriched in the cooler treatment, whereas PROT1 and CHRY1 were enriched in the warmer treatment (Fig. S4). Although total variation attributed to the dispersal treatment was low (Table S3), high dispersal between mesocosms, i.e. without filtering, facilitated the enrichment of some taxa; for instance, ASAI1/2 and STEN1. In contrast, STEN2 and PROT1 were slightly more enriched in the low dispersal treatment.

### *Chryseobacterium* and *Pseudomonas* strains influence *Ae. albopictus* development

**Figure 4:**
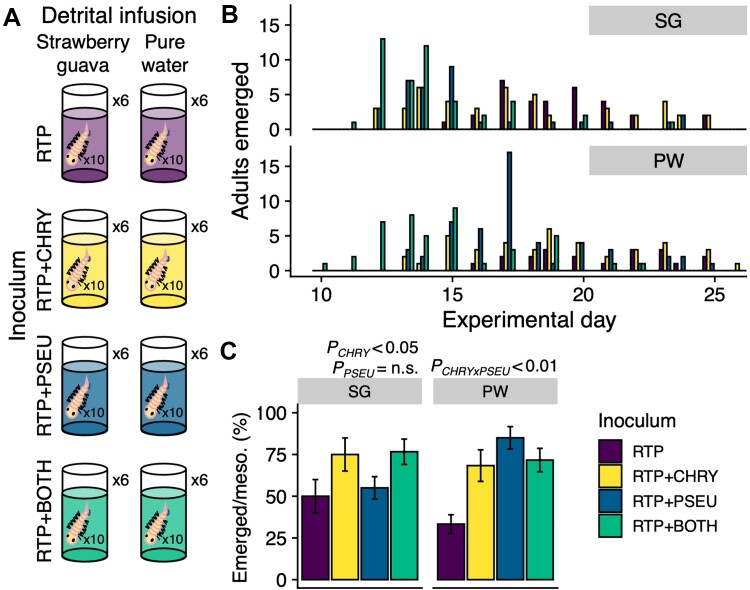
**Overview of experiment II: Mosquito development.** (**A**) Mesocosms with each of eight factorial treatments: Two infusions (SG: strawberry guava, PW: pure water) crossed with four inocula (RTP: The RTP-II base, +CHRY: Addition of *Chryseobacterium* sp., +PSEU: Addition of *pseudomonas* sp., +BOTH: both *pseudomonas* sp. and *Chryseobacterium* sp.). Each mesocosm had six replicates and contained 10 *Ae. albopictus* larvae each (48 mesocosms, 480 larvae total). (**B**) number of adult mosquitoes that emerged per experimental day, colored by inoculum, and arranged vertically by infusion. (**C**) bars indicate the average percentage of larvae that emerged as adults per mesocosm, with error bars displaying the standard error. The bottom right legend applies to all panels.

Emergence success was higher across Experiment II than Experiment I, with 313 adults emerging from 480 larvae placed into mesocosms (65.2%). In SG mesocosms, the availability of CHRY1 in the species pool increased the probability of emergence as an adult (*P* < 0.01), whereas including PSEU2 did not (*P* = 0.69) (Fig. 4). In PW mesocosms, including PSEU2 alone increased emergence success the most over the base RTP-I taxa (Fig. 4). However, there was a significant interaction with CHRY1 (*P* < 0.01) which caused emergence rates with both taxa to better match CHRY1-only emergence rates than PSEU2-only emergence rates (Fig. 4).

Both CHRY1 and PSEU2 decreased the development time of the larvae that successfully emerged as adults in SG (*P* < 0.05 and *P* < 0.001, respectively) and PW mesocosms (*P* < 0.01 and *P* < 0.001, respectively) (Figs. 4, S11). The effect sizes were generally larger in both infusion types for PSEU2 (Cox hazard ratios of 3.5 and 3.1 for SG and PW mesocosms, respectively) when compared to CHRY1 (Cox hazard ratios of 1.6 and 2.1 for SG and PW mesocosms, respectively) (Figs. 4, S11). There was no significant interaction between CHRY1 and PSEU2 for development time in either infusion type.

## Discussion

We examined the impacts of abiotic (leaf detrital infusion, temperature) and biotic (microbial dispersal) larval habitat conditions on adult mosquito emergence and their associated microbiomes. Infusions’ interactions with temperature were the most significant drivers of whether and how quickly mosquitoes emerged (Fig. 1). Regarding bacterial community composition within adults, infusion consistently explained more variation than temperature or dispersal regimes (Fig. 2, Table S2). Bacterial taxa displayed distinct patterns, including a higher relative abundance of strain CHRY1 in the strawberry guava-reared adults and higher PSEU2 in the pure water-reared adults (Fig. 3). Our follow-up experiment indicated that manipulating the presence of these two strains impacted larval development success across infusions (Fig. 4), bridging microbes’ relative abundance differences to host fitness implications. Together, our results indicate that environmental context plays a defining role in the outcomes of host–microbe interactions.

### Botanical detritus infusion and temperature influence *Ae. albopictus* development

The fastest and highest rates of mosquito emergence occurred from larval mesocosms with the Hawaiian native plant infusion (‘ōhi‘a lehua; OL) when compared to both the invasive plant infusion (strawberry guava; SG) and pure water (PW) control (Figs. 1, S1). All infusions were filtered during setup, which could have allowed minute particulate matter, dissolved nutrients, and small microorganisms (e.g. viruses, small bacteria) to enter the experiment. As we homogenized and re-allocated a small amount of the water column between mesocosms to simulate microbial dispersal, incidental microorganisms introduced through the non-sterile detrital infusions could have been established across all treatments. However, the total bacterial community compositions across infusions remained distinct (Fig. 2), were dominated by taxa matching the RTP-I cultures (> 96% relative abundance of non-*Wolbachia* reads; Fig. S3), and no bacteria aside from those in the RTP-I varied across infusions (Fig. S6). Components of the OL infusion may have benefitted the mosquito larvae directly *via* larval consumption or indirectly through metabolic products from aquatic microorganisms. Our study would have benefitted from a closer examination of aquatic chemistry, e.g. in terms of nitrogen, phosphorus, and carbon. The same dry mass of OL and SG was provided, which hints that the native plant type conferred a unique benefit. Alternatively, SG has allelopathic traits [45], which may have negatively impacted the mosquito larvae and/or microbes or countered any benefits of the botanical infusion. Other studies have found no relationship or a complementary one between invasive plant detritus with mosquito population metrics [46–48]. Thus, it appears consistent that local plant assemblages can affect mosquito population dynamics, although specific outcomes are context-dependent.

Temperature also influenced mosquito emergence, with mosquitoes emerging faster in warmer mesocosms for all three infusion types (Figs. 1, S1). This finding is consistent with an increased metabolic rate under warmer temperatures, when thermal limits are not exceeded, and has been observed previously in *Ae. albopictus* [49, 50]. In prior work, when examining only the influence of temperature, mosquitoes emerged faster in warmer temperatures, but the total number of adults was typically lower than at cooler temperatures [49, 50]. Our pure water mesocosms corroborate this finding, but crossing temperature with botanical infusions largely weakened differences in the number of adults (Fig. 1). Future examinations of aquatic chemistry differences across detrital infusions would help shed light on possible mechanisms underlying this result. In sum, the detrital infusion-temperature interactions found here highlight the importance of evaluating interactions between habitat quality and climate change for disease vector organisms.

### Subset of larval habitat microbes persist in adult mosquitoes

All but one of Experiment I’s inoculated bacteria were identified in the mesocosm water samples, indicating they were likely available to mosquito larvae; yet, there were distinct patterns in their persistence among adult mosquitoes (Fig. 3, Table 1). These bacteria were originally isolated from adult mosquito midguts and have been observed in other local studies [10, 25–27], thus they presumably exist in a level of symbiosis in nature. However, mosquitoes purge most microbes during metamorphosis [28, 51]. The current study design does not allow us to distinguish whether the inoculated bacteria were lost during metamorphosis or never established within these mosquitoes. As adults emerged separately in sterile water, we can conclude that the bacteria sequenced in adults were retained from earlier life stages. We noted distinct differences in which inoculated bacteria persisted among adult mosquitoes, even among closely related taxa. For instance, Experiment I included multiple cultures from the *Pseudomonas* and *Pantoea* genera, but only one culture from each genus appeared within adult mosquitoes (Fig. 3, Table 1). Moreover, examining microbial compositions across abiotic factors, patterns did not align with phylogenetic relatedness; each ASV demonstrated unique responses (Fig. S9). More in-depth sequencing and assays than the 16S rRNA gene would be required to better examine taxonomic or functional differences between these cultured bacteria. Regardless, the pattern of nestedness from a more diverse environment to a less diverse host is well supported in mosquito-based and environmental studies [10, 51, 52]. Similarly, we can conclude that the microbial diversity across the artificial environments here does not reliably predict similar patterns in adult mosquitoes, even among closely related bacterial taxa, suggesting a role of the host and/or its resident microbes in regulating establishment.

One consistent pattern between the free-living and host-associated bacterial communities is that detrital infusion accounted for the most variation. A previous study manipulated high and low concentrations of nutrients (alfalfa pellets) and found no differences in total microbial community composition in *Culex nigripalpus* mosquitoes [53]. In synthesizing our two studies, a provided resource’s specific chemical makeup may have a more stark impact on microbiota than its concentration. In addition, our study provided the botanical detritus’ leachate rather than the source itself. Despite a lack of overarching community-level changes, the previous study did find an enrichment of specific taxa across treatments, including *Clostridiales* in the high-nutrient scenario and *Burkholderiales* in the low-nutrient scenario [53]. We did not examine these bacterial orders in our experiment, but we applied a mixed model framework to identify the taxa with the most consistent differences across detrital infusion treatments. The two taxa with the highest likelihood of observing in mosquitoes, and comparatively lower likelihood of encountering in their larval water, were strains of *Chryseobacterium* sp. and *Pseudomonas* sp. in the SG and PW treatments, respectively (Figs. 3, S5).

### Environment and microbe pairings influence mosquito development

Whereas results from Experiment I were largely correlational, in Experiment II the specific presence of two bacterial strains (CHRY1 and PSEU2) impacted *Ae. albopictus* developmental success (Fig. 4). Although the majority of mosquito-associated microbes have not yet been ascribed specific functions, both *Chryseobacterium* and *Pseudomonas* are commonly found in the gut communities of *Aedes* spp. [7, 13, 54], suggesting that they may be important symbiotic partners. Furthermore, both genera have been identified during critical stages of reproduction and continuation of the mosquito life cycle. For instance, *Pseudomonas aeruginosa* helped attract *Ae. aegypti* mosquitoes for oviposition [55] and a labeled *Pseudomonas* isolate was traced through multiple life stages of malaria vector *Anopheles stephensi* [56]. *Chryseobacterium* was previously identified through multiple life stages of *Ae. aegypti* and was one of six microbes that rescued the development of axenic larvae [7]. Two putative *Pseudomonas* spp. were identified as core taxa (present in > 90% of samples) in a common garden experiment with three different mosquito species, including *Ae. albopictus* [12]. Additional recent work, including microbiome transplantation, indicates that mosquito-bacteria associations may not be highly specialized to specific host species [11, 12, 57]. These findings may be explained by generalized functional redundancy across many commonly associated bacteria, which should be examined further through direct assays and/or genomic profiling. Nevertheless, our findings contribute to the body of evidence that genera *Chryseobacterium* and *Pseudomonas* contain isolates that are commonly mosquito-associated, impact development, and warrant further inquiry.

In line with relative abundance differences in Experiment I (Fig. 3), the effect of CHRY1 on the proportion of adults that emerged was more pronounced in the SG environment, and the effect of PSEU2 was more pronounced in the PW environment in Experiment II (Fig. 4), suggesting unique microbe by environment interactions. Specifically, including CHRY1 in the inoculum with SG infusion resulted in higher emergence, regardless of whether PSEU2 was present (Fig. 4). In the PW environment, CHRY1 supported emergence success over the base inoculum, but not to the level that PSEU2 achieved without CHRY1 (Fig. 4). This raises many potential avenues of future investigation; first, CHRY1 can provide support for emergence success in multiple environmental contexts, i.e. it may be a symbiont that provides generalizable functions across environments. In contrast, PSEU2 may be specialized to support development under depauperate conditions with fewer resources and interacting microbes. In addition, the significant interaction between CHRY1 and PSEU2 in the PW environment hints at potential microbe competition. Accordingly, network-based evidence identified *Pseudomonas* as a hub taxa in *Ae. albopictus*, including both positive and negative correlations with other bacteria [54]. Although we reduced the microbial community members between RTP-I and RTP-II from 23 taxa to six, reducing the potential for complex interactions, CHRY1, or PSEU2 interactions with any or all of the six taxa of RTP-II may have contributed to the observed patterns. Conditions during earlier developmental stages, such as nutrient provisioning, could have provided carryover benefits that persisted into later stages (e.g. [19]) and co-varied with intra-adult bacterial abundance. Future study is required to resolve the mechanisms that confer such changes, yet our data suggest that the presence of specific microbes across different environments modulates a fundamental parameter of host fitness.

## Conclusions

Our cross-factorial design emulated interacting real-world stressors that currently affect biological disease vectors. Specifically, our results showed warmer temperatures encouraged faster mosquito development, which aligns with ongoing range expansion and population growth of mosquitoes in some tropical and subtropical environments and the spread of associated diseases [58, 59]. *Ae. albopictus* is an invasive mosquito in Hawai‘i and we found poor developmental success when reared with strawberry guava-infused water, which displays a negative interaction between invasives that warrants further research. Microbiome acclimation offers a new dimension to increase the competitiveness of lab-reared mosquitoes in nature [6] and more generally aids in understanding host performance among diverse or changing environmental landscapes [60]. Our data offer further support that manipulating specific microbial taxa can confer advantages but also show that the nature and efficiency of these relationships vary with the environment.

## Supplementary Material

Mosqmicrobes_supps_revising_wrae181

table_s1_rtp_info_wrae181

## Data & Analysis Availability

Raw sequencing reads are available at the National Center for Biotechnology Information’s Short Read Archive (PRJNA1125914). Additional data and scripts for analysis are available at github.com/nicfall/mosquito_microbes/.
