# Supplementary material for: Abiotic factors shape mosquito microbiomes that enhance host development
Source: ISME J. 2024 Sep 24;18(1):wrae181. doi: 10.1093/ismejo/wrae181 (PMC11481732; doi:10.1093/ismejo/wrae181)
Supplement: Mosqmicrobes_supps_revising_wrae181 [file mosqmicrobes_supps_revising_wrae181.docx]

**Abiotic factors shape mosquito microbiomes that enhance host development**

Running head: Mosquito microbiomes among environments

Kriefall, Nicola G.^1^*, Seabourn, Priscilla S.^1^, Yoneishi, Nicole M.^1,2^, Davis, Kahiwahiwa^1^, Nakayama, Kirsten K.^1,2^, Weber, Danya E.^1^, Hynson, Nicole A.^1,2^, Medeiros, Matthew C. I.^1,2^

*corresponding author: Nicola G. Kriefall, Pacific Biosciences Research Center, University of Hawai‘i at Mānoa, 1993 East-West Road, Honolulu, HI 96822, USA. nicolagk@hawaii.edu

^1^Pacific Biosciences Research Center, University of Hawai‘i at Mānoa, 1993 East-West Road, Honolulu, HI 96822, USA

^2^Center for Microbiome Analysis through Island Knowledge and Investigation, University of Hawai‘i at Mānoa, 1993 East-West Road, Honolulu, HI 96822, USA

### SUPPLEMENTARY METHODS

*Preparation of botanical detritus infusions*

Infusions were made by mixing 3.2 g of plant material (90% senesced and 10% green leaves) in 160 mL of sterile water and incubating the mixture for 5 days at a 12:12 hour 27.5^o^C diurnal/23.5^o^C nocturnal cycle with a relative humidity of > 60%. Three replicate infusions were made for each tree species. Infusions were double-filtered (with a 0.2 µm filter followed by a 0.1 µm filter) and diluted at a 1:6 ratio with sterile water in each mesocosm. Each replicate infusion provided aquatic media for eight mesocosms, evenly partitioned across the experimental treatment levels.

*Isolation of mosquito-associated bacteria*

​Mosquito midguts were isolated from mosquitoes that were surface sterilized with 75% ethanol for 1 minute and rinsed twice in sterile 1X phosphate-buffered saline (PBS). Midguts were dissected using sterile forceps (Bioquip, Rancho Dominquez, CA) before being placed in 80 µL of 1X PBS and homogenized with 1.4 mm ceramic beads at 2.1 m/s for 30 s using a Bead Ruptor 24 (OMNI International, Kennesaw, GA). Homogenized midguts were immediately placed in LB broth and shaken at 480 RPM at 28^o^C for 24 hours. After 24 hours, the LB broth was checked for confluency and plated on either LB, blood, or nutrient agar plates at a 10^-3^ dilution and grown at 28^o^C for 24 to 48 hours. Individual colonies were isolated and re-plated to establish pure cultures. Pure isolated colonies were preserved in 25% glycerol and kept at -80^o^C until further processing. Bacterial colonies were identified taxonomically and their purity confirmed using Sanger sequencing of the V4 region of the 16S rRNA gene (Table S1). Primers and PCR conditions for Sanger sequencing were equivalent to preparation of 16S rRNA amplicon libraries (see below).

*Preparation of 16S rRNA amplicon libraries*

DNA was extracted from the aquatic media and adult mosquito samples, which had been surface sterilized by a 75% ethanol wash, followed by two rinses in sterile 1X PBS. Three negative controls with sterile water in lieu of a sample were also prepared. DNA was extracted and purified using a Macherey-Nagel NucleoMag Tissue kit (Macherey-Nagel GmbH & Co. KG, Düren, Germany) on a KingFisher Flex (Thermo Fisher Scientific, Waltham, MA) following the manufacturer’s protocol. Library preparation was done using a modified version of the Earth Microbiome Project 16S V4 Illumina Amplicon Protocol. Standard Earth Microbiome Project barcoded 16S primers were used, based off of 515F (5’-GTGYCAGCMGCCGCGGTAA-3’) and 806R (5’- GGACTACNVGGGTWTCTAAT-3’) primers [[1, 2]](https://paperpile.com/c/cOJawy/UntXw+xhCWw). Each PCR amplification contained the following components: 16.25 µL of nuclease-free water, 5.0 µL of 5X KAPA HiFi Fidelity Buffer, 1.0 µL of template DNA (approximately 10-87 ng), 0.75 µL of 10 mM KAPA dNTP Mix, 0.75 µL of 10 µM forward primer, 0.75 µL of 10 µM reverse primer, and 0.5 µL of 1 U/µL KAPA HiFi HotStart DNA Polymerase. Two no-template PCR amplifications were also performed as additional negative controls. PCR amplifications were performed in an Applied Biosystems SimpliAmp Thermal Cycler (Thermo Fisher Scientific, Waltham, MA) under the following conditions: initial denaturation at 95^o^C for 3 min; 35 cycles of denaturation at 98^o^C for 20 s, annealing at 60^o^C for 15 s, extension at 72^o^C for 30 s; and a final extension at 72^o^C for 30 s. The PCR products were visualized on a 2% agarose gel before purification and normalization to approximately 1.25 to 2.50 ng/µL using a Just-a-Plate kit (Charm Biotech, Cape Giradeau, MO). Purity and concentration of a subset of samples was assessed with a NanoDrop (Thermo Fisher Scientific, Waltham, MA). A volume of 10 µL from each sample, including the five negative controls, was pooled and then purified and concentrated using a 1.2X volume of homemade AMPureXP Beads.

Wolbachia *qPCR procedures*

Total *Wolbachia* sp. load was quantified using qPCR with *Wolbachia*-specific primers (W-Spec-16S-F: 5’-CATACCTATTCGAAGGGATA-3’ and W-Spec-16S-R: 5’-AGCTTCGAGTGAAACCAATTC-3’) [[3]](https://paperpile.com/c/cOJawy/sRUWu). Primers of the actin gene, alb-act-F (CCCACACAGTCCCCATCTAC) and alb-act-R (CGAGTAGCCACGTTCAGTCA) [[4]](https://paperpile.com/c/cOJawy/kHe1y), were used to quantify host genomic copies. Each qPCR amplification contained the following components: 5.0 µL of 2X PowerUp SYBR Green Master Mix (Applied Biosystems, Foster City, CA), 1.0 µL of nuclease-free water, 1.0 µL of 10 µM forward primer, 1.0 µL of 10 µM reverse primer, and 2.0 µL of template DNA (approximately 10-87 ng). The reactions were performed using an Applied Biosystems StepOne Plus Real-Time PCR System (Thermo Fisher Scientific, Waltham, MA) under the following conditions: UDG activation at 50^o^C for 2 min; Dual-Lock DNA polymerase at 95^o^C for 2 min; and 40 cycles of denaturation at 95^o^C for 15 s and annealing/extension at 60^o^C for 1 min. Fluorescence readings were taken at the 60^o^C annealing/extension step for each cycle. A melt curve stage was performed according to the PowerUp SYBR protocol to confirm the specificity of amplification. The Cycle threshold (Ct) values were used to estimate load intensity and were obtained assuming a delta Rn fluorescence threshold of 0.3. The relative abundance (i.e., *Wolbachia* index) was estimated as a ratio of the inverse Wolbachia Ct value to the inverse Ct of the single copy mosquito gene, actin. Any qPCR samples with no detected Wolbachia template received a Ct value of 41 (total cycles in the reaction plus one).

*Experiment I bioinformatics for microbiome composition*

Two SG water samples from day four failed during sequencing, leaving 214 mesocosm water samples. Average (± s.d.) raw 16S rRNA amplification reads per sample were 48,889 (± 17,494) for mosquitoes and 31,083 (± 14,527) for mesocosm water samples. Average reads per sample post-processing were 42,510 (±17,504) for mosquitoes and 27,418 (±12,733) for mesocosm water. Average raw reads for the botanical infusion and RTP-I samples were: 19,604 (± 4,514) for OL; 7,129 (± 2,755) for SG; 4,517 (± 2,069) for SW; and 42,763 for RTP-I (no replicate samples for calculating s.d.). Post-processing, these averages became: 14,739 (± 3,019) for OL; 4,896 (± 2,023) for SG; 1,474 (± 676) for SW; and 35,051 for RTP-I.

Removal of 68 potential contaminant ASVs from the total dataset was carried out using *decontam* [*[5]*](https://paperpile.com/c/cOJawy/agZtT). One ASV identified as a contaminant and present in one out of five negative controls was retained for analysis as it matched an intended strain in the RTP-I (CARN1; Figure S2). Three mesocosm water samples with less than 9,200 reads, the minimum read count used for rarefaction below, were removed from analysis (one from each of an OL mesocosm and SG mesocosm on day 12 and one from a PW mesocosm on day 20). In addition, two mosquito samples below this read count threshold were removed (one male and one female from OL mesocosms). ASVs present in only one sample or with less than 153 reads across all samples (*i.e.* less than 0.001% of total reads) were removed.

The following analyses were conducted on both the > 3.5% prevalence dataset and full dataset. However, patterns from the two datasets were equivalent and only results from the > 3.5% prevalence dataset are presented in order to facilitate comparison to GLMM results. Packages *phanghorn* [*[6]*](https://paperpile.com/c/cOJawy/WC0jj) and *DECIPHER* [*[7]*](https://paperpile.com/c/cOJawy/srakY) were used to calculate phylogenetic distances between taxa. ASV counts were aggregated by mesocosm to account for lack of independence of larvae from the same mesocosm. Weighted UniFrac and Aitchison distances were calculated using *vegan* [[8]](https://paperpile.com/c/cOJawy/WV40O). Statistical differences in both weighted UniFrac and Aitchison distances between experimental groups was assessed using *adonis2* in package *vegan* [[8]](https://paperpile.com/c/cOJawy/WV40O) with fixed effects of infusion, temperature, dispersal. In addition, the *betadisper* function in *vegan* [[8]](https://paperpile.com/c/cOJawy/WV40O) informed whether significant results were due to heterogeneity of variances across groups.

### SUPPLEMENTARY RESULTS

Wolbachia *qPCR results*

The index of absolute *Wolbachia* sp. abundance did not vary significantly with infusion type (*P* = 0.68), temperature (*P* = 0.59), time period of emergence (*P* = 0.13), or dispersal (*P* = 0.63). The effect of sex on this *Wolbachia* sp. index was marginally insignificant (*P* = 0.09), with males having slightly greater *Wolbachia* sp. abundance than females.

*Mesocosm water alpha diversity in Experiment I*

During initial mesocosm setup, mean ASV richness (± s.d.) of water samples from the three replicate infusions was 45.7 (± 3.2) for OL, 23.7 (± 6.4) for SG, and 4.3 (± 1.2) for PW. The majority (59%) of these ASVs were absent for the remainder of the experiment, indicating they were likely DNA from non-viable cells during setup. When examining ASV richness across treatments, there was a significant interaction between day of sample collection and infusion type (*P* < 0.001; Figure S8). Richness was largely homogenous across infusions on day 4, while OL mesocosms were highest on average, followed by SG, and finally PW on days 12 and 20 (Figure S8). Mean water richness was independent of both dispersal (*P* = 0.38) and temperature (*P* = 0.38) treatments. Simpson's index (inverted) also displayed an infusion by day interaction (*P* < 0.001), where values became more similar across infusions by day 20 (Figure S8). On days 4 and 12, PW mesocosms had the highest Simpson’s index, followed by OL, and lastly SG (Figure S8). Temperature also influenced Simpson’s index, where warmer mesocosms were higher on average (*P* < 0.001; Figure 2), but there was no effect of dispersal (*P* = 0.84). With rarefied data, relative differences in effect sizes across groups and ascribed levels of statistical significance held for both ASV richness and Simpson’s index.

**REFERENCES**

1. [Parada AE, Needham DM, Fuhrman JA. Every base matters: assessing small subunit rRNA primers for marine microbiomes with mock communities, time series and global field samples. *Environ Microbiol* 2016; **18**: 1403–1414.](http://paperpile.com/b/cOJawy/UntXw)

2. [Apprill A, McNally S, Parsons R, Weber L. Minor revision to V4 region SSU rRNA 806R gene primer greatly increases detection of SAR11 bacterioplankton. *Aquat Microb Ecol* 2015; **75**: 129–137.](http://paperpile.com/b/cOJawy/xhCWw)

3. [Werren JH, Windsor DM. Wolbachia infection frequencies in insects: evidence of a global equilibrium? *Proc Biol Sci* 2000; **267**: 1277–1285.](http://paperpile.com/b/cOJawy/sRUWu)

4. [Calvitti M, Marini F, Desiderio A, Puggioli A, Moretti R. Wolbachia density and cytoplasmic incompatibility in Aedes albopictus: concerns with using artificial Wolbachia infection as a vector suppression tool. *PLoS One* 2015; **10**: e0121813.](http://paperpile.com/b/cOJawy/kHe1y)

5. [Davis NM, Proctor DM, Holmes SP, Relman DA, Callahan BJ. Simple statistical identification and removal of contaminant sequences in marker-gene and metagenomics data. *Microbiome* 2018; **6**: 226.](http://paperpile.com/b/cOJawy/agZtT)

6. [Schliep KP. phangorn: phylogenetic analysis in R. *Bioinformatics* 2011; **27**: 592–593.](http://paperpile.com/b/cOJawy/WC0jj)

7. [Wright E. Using DECIPHER v2.0 to analyze big biological sequence data in R. *R J* 2016; **8**: 352.](http://paperpile.com/b/cOJawy/srakY)

8. [Oksanen, Kindt, Legendre, O’Hara. The vegan package. *Community Ecol* .](http://paperpile.com/b/cOJawy/WV40O)

9. [Dahl EM, Neer E, Bowie KR, Leung ET, Karstens L. microshades: An R Package for Improving Color Accessibility and Organization of Microbiome Data. *Microbiol Resour Announc* 2022; **11**: e0079522.](http://paperpile.com/b/cOJawy/rIKt)

10. [Lenth RV. Estimated Marginal Means, aka Least-Squares Means [R package emmeans version 1.8.2]. 2022.](http://paperpile.com/b/cOJawy/233oE)


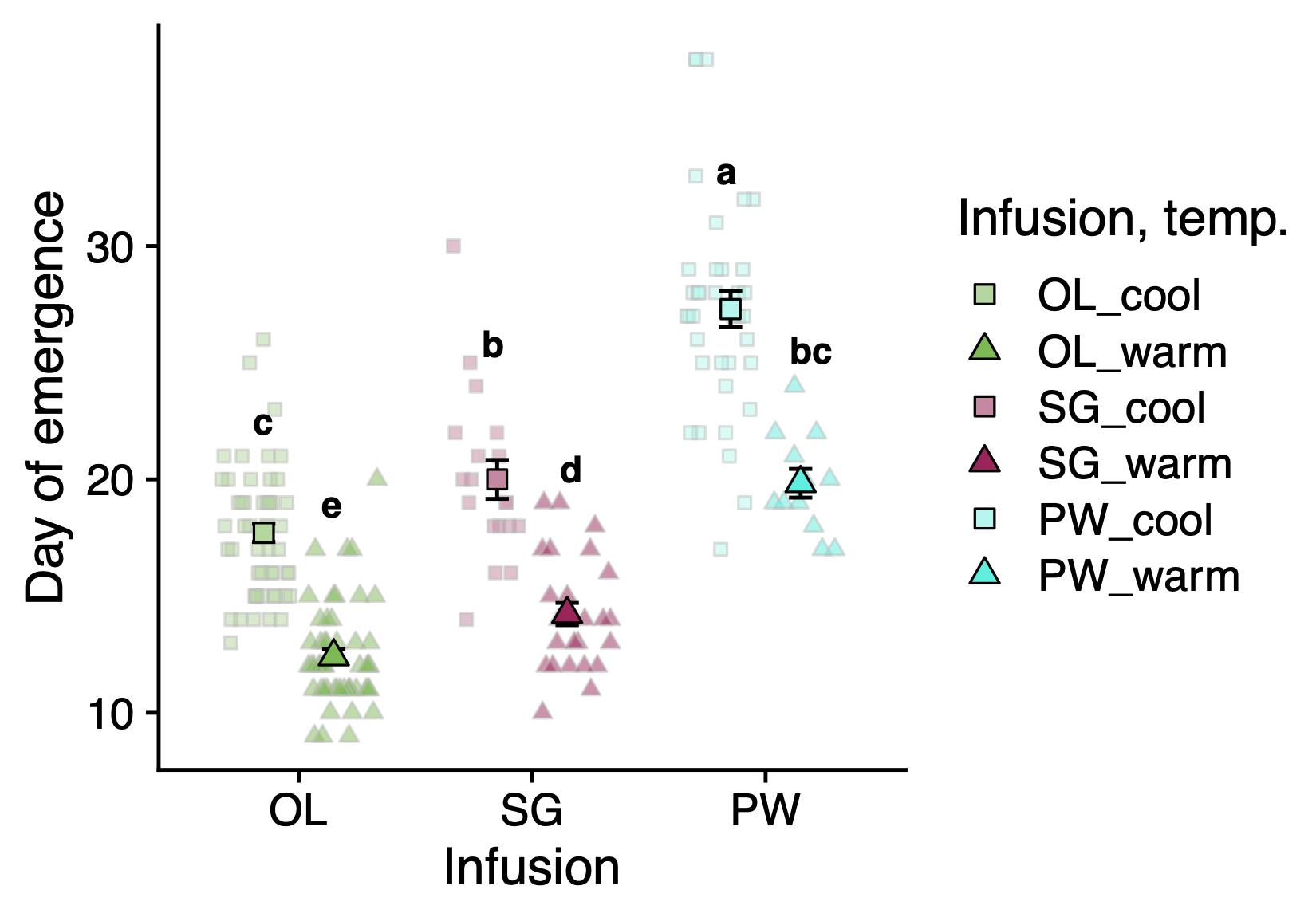


### Figure S1. Day of emergence of adult *Aedes albopictus* compared between infusion types (OL: ‘ōhi‘a lehua, SG: strawberry guava, PW: pure water) and temperature (cool: squares, warm: triangles) treatments. Central symbols represent the mean, with error bars representing standard error. Different letters above the symbols indicate a significant difference in Tukey test *post hoc* comparisons based on package *emmeans* (*p*<0.05; [9]).

### Table S1. Information on each of 23 RTP-I bacterial cultures, which were originally isolated from adult female mosquito midguts. The “FASTA” column provides the 515-806 V4 region of the 16S rRNA gene sequenced from the cultures *via* Sanger sequencing. All culture sequences were aligned with the ASVs found in the RTP-I sample: “Percent identity” provides the percent identity with the matching ASV, whose ID is provided as “Equivalent ASV”. Lastly, “RTP-I rel. abundance” provides the percentage of reads that each culture comprised within the RTP-I sample. This column adds up to 96.3% rather than 100% as it excludes ASVs that matched *Wolbachia* or had no clear taxonomic match (*i.e.* < 99.60% percent identity) with the cultured strains.

(Attached in separate document)


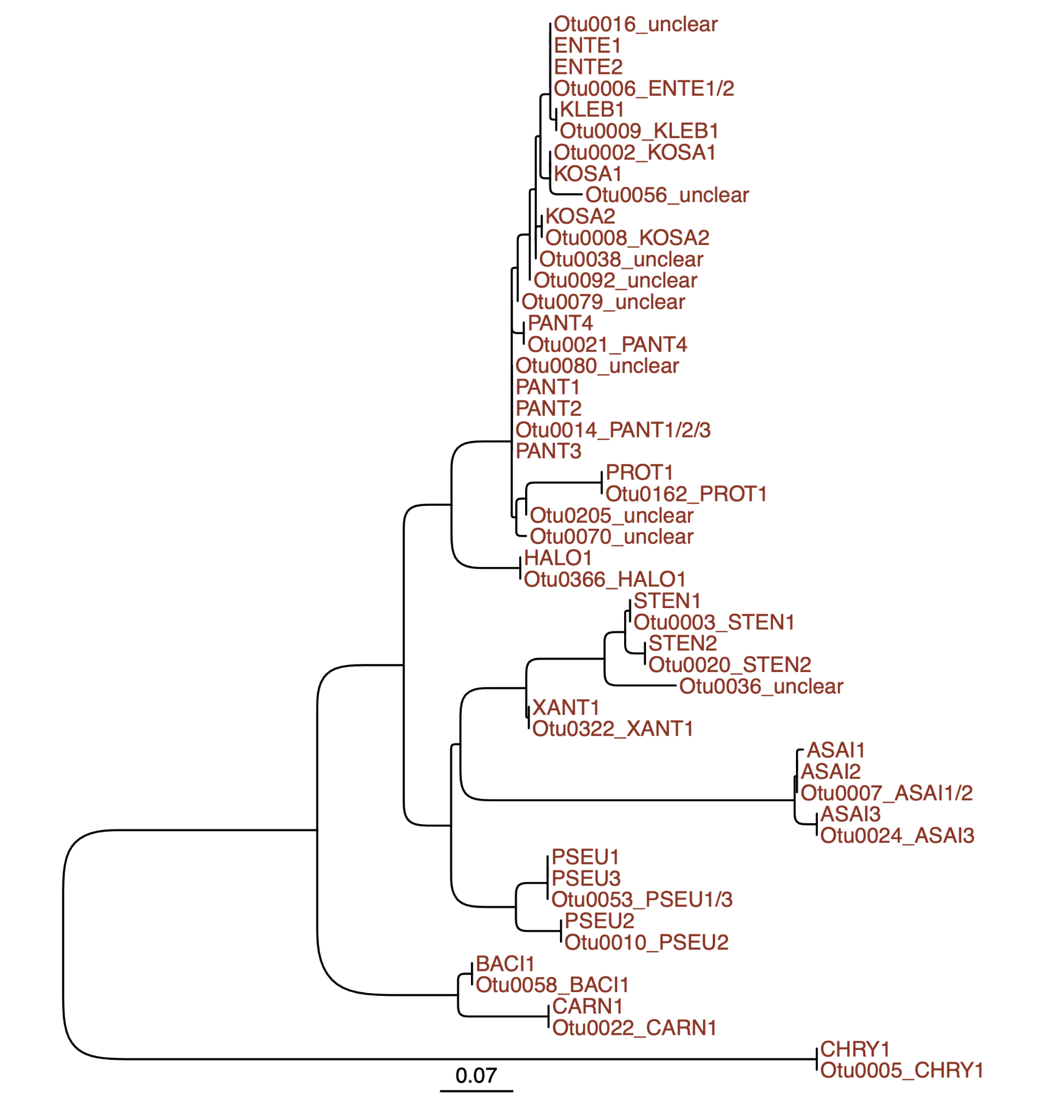


### Figure S2. Phylogenetic tree displaying distances between ASVs of the current experimental dataset (names beginning with “Otu”) and Sanger sequencing data of the 16S rRNA gene of cultured bacteria used for the RTP-I inoculum. ASVs were either named by their matching culture or labeled “unclear” if no match was found (*i.e.* <99.60% percent identity).

###
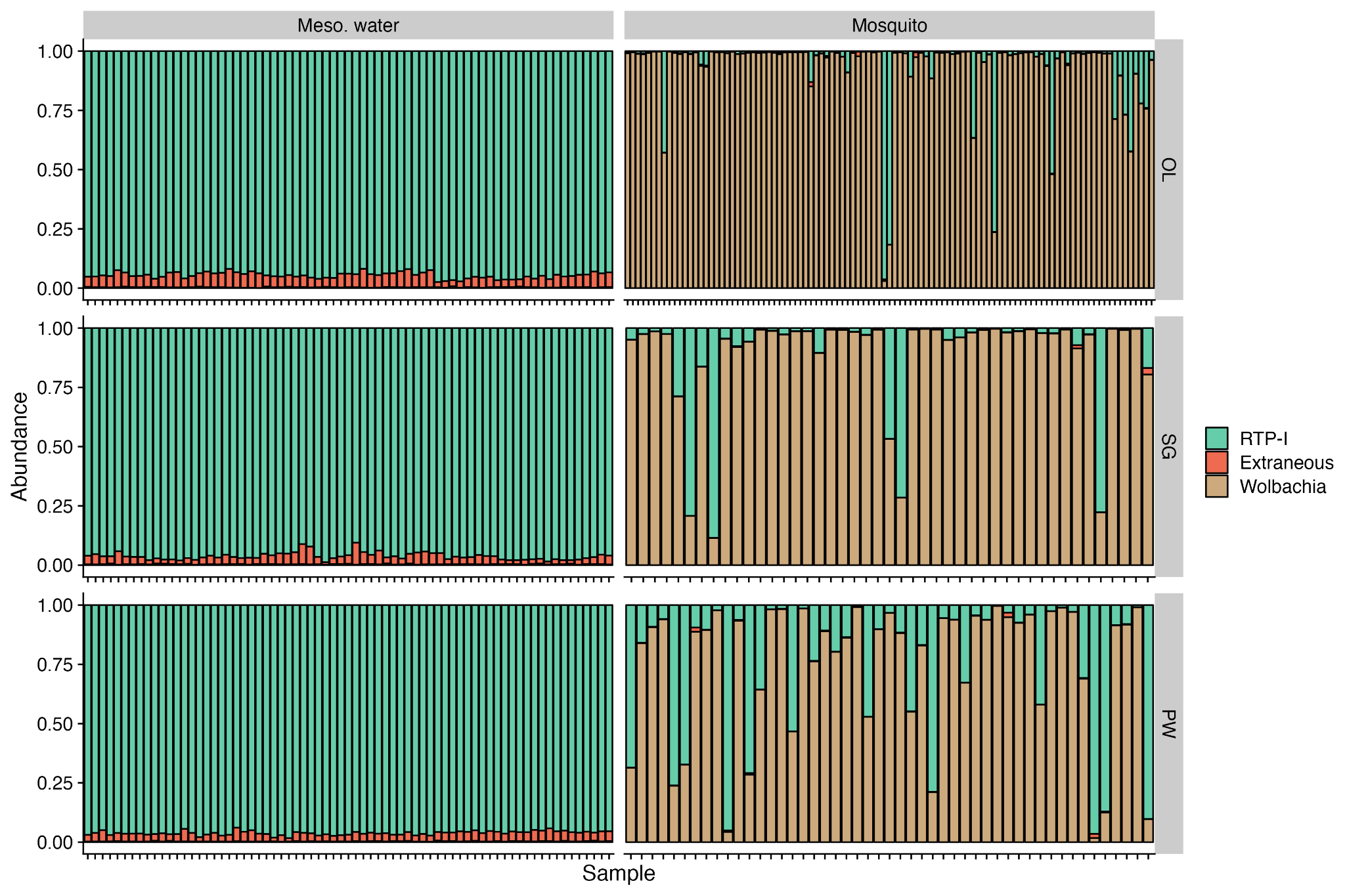
Figure S3. Relative abundance of bacterial taxa in mosquitoes (left) and mesocosm water (right). Panels are arranged vertically by infusion (OL: ‘ōhi‘a lehua, SG: strawberry guava, PW: pure water). Each bar represents a sample, where the colors indicate the relative abundance of reads sourced from the “RTP-I” (*i.e.* known cultured taxa), “Extraneous” (*i.e.* undetermined bacterial diversity), or from the genus *Wolbachia*.

### Table S2. Results from three statistical comparisons of microbiome community composition of the taxa with >3.5% prevalence in mosquitoes across experimental variables. For the Generalized Linear Mixed Model (GLMM) results, the variance (± standard deviation) of the random effects’ conditional models is presented. For Aitchison distance and weighted UniFrac metrics, the *R*^2^ is given. Each of these results is followed by the associated *P* value and level of significance. “NA” indicates an inability to assess a variable due to aggregation of data by mesocosm identity. “^” indicates significant beta dispersion differences between groups, which may qualify the significance of the multivariate analysis.

| **Variable** | **GLMM** | **Aitchison distance** | **Weighted UniFrac** |
| --- | --- | --- | --- |
| Infusion | 2.14 (±1.46)  *P*<0.001*** | *R*^2^=0.16  *P*<0.001*** | *R*^2^=0.21  *P*<0.001***(^) |
| Temperature | 0.24 (±0.49)  *P*=0.054 | *R*^2^=0.03  *P*<0.05* | *R*^2^=0.04  *P*<0.05* |
| Dispersal | 0.09 (±0.71)  *P*=0.25 | *R*^2^=0.02  *P*=0.07 | *R*^2^=0.02  *P*=0.24 |
| Sex | 0.26 (±0.51)  *P*<0.001*** | NA | NA |
| Time period | 0.26 (±0.51)  *P*<0.05* | NA | NA |

###

###

###

###
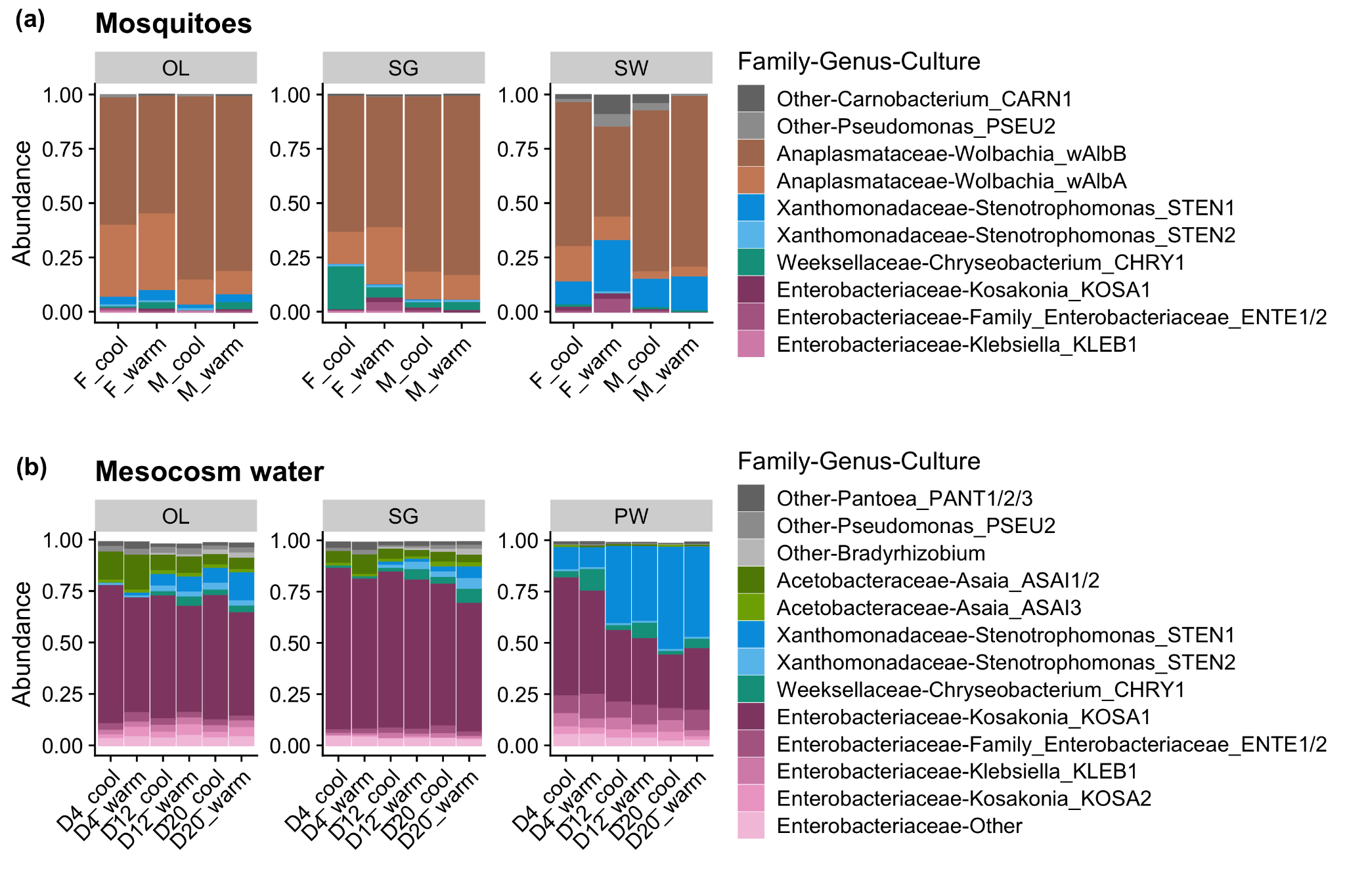


### Figure S4. Relative abundance of bacterial taxa in (a) mosquitoes and (b) mesocosm water. Panels are arranged horizontally by infusion (OL: ‘ōhi‘a lehua, SG: strawberry guava, PW: pure water). (a) Sample sums of female mosquitoes begin with “F” and males with “M”, followed by the temperature treatment (cool or warm). (b) Sample sums of mesocosm water samples begin with the day number (“D” followed by 4, 12, or 20) and are followed by the temperature treatment. For all panels, bacterial taxa names consist of the following: family, genus, and the shorthand names of RTP-I bacteria, if applicable. Taxonomic classifications that were not in the top 4 most abundant of its kind were labeled as “Other”. Lower abundance taxa not clearly visible were excluded for visualization. Plot generation was facilitated by package *microshades* [[9]](https://paperpile.com/c/cOJawy/rIKt).

###

###

###
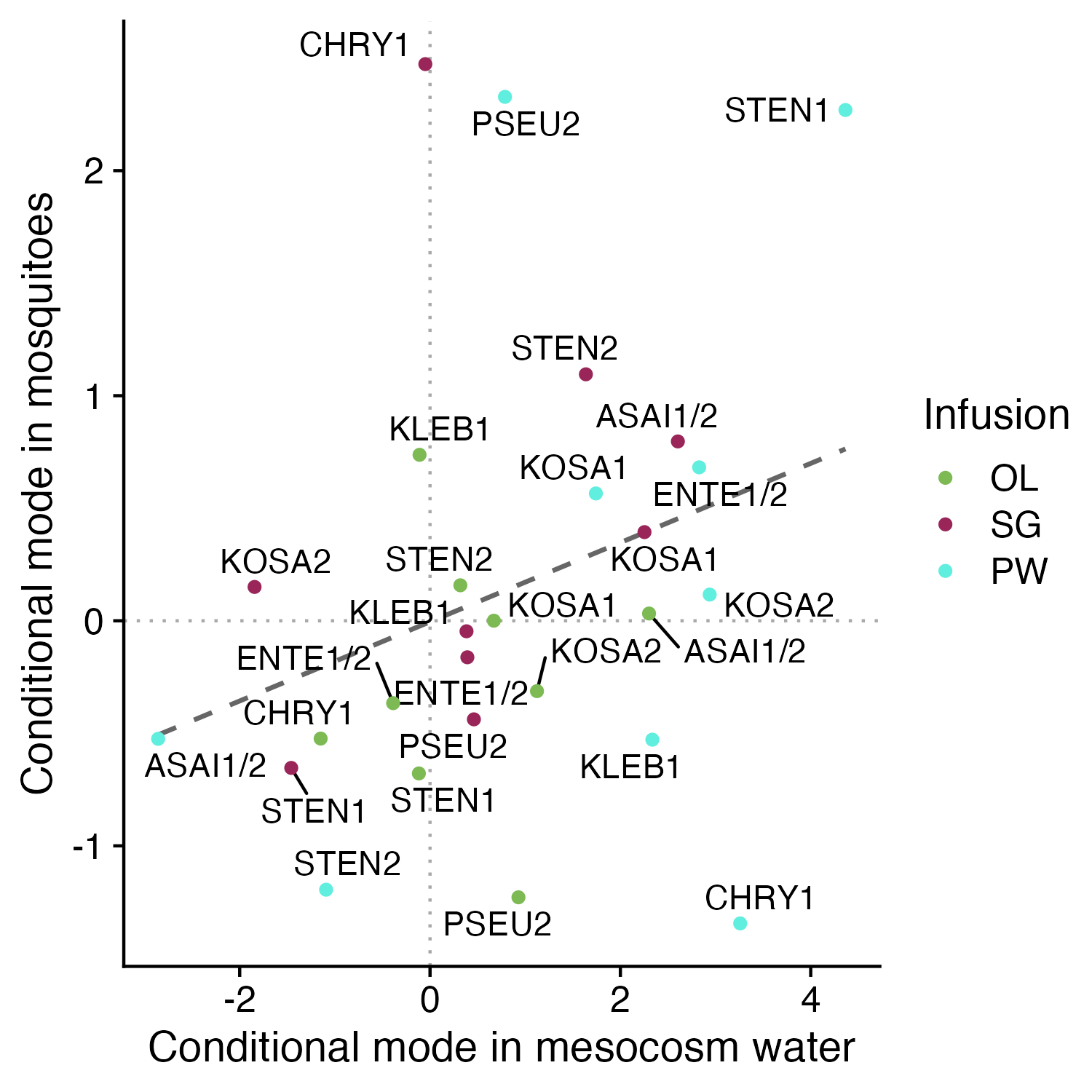


### Figure S5. Conditional modes of inoculated bacteria in the mosquitoes (y-axis) compared to the mesocosm water (x-axis). ASVs are labeled by their respective matching culture name. Colors of the dots represent the infusion type (OL: ‘ōhi‘a lehua, SG: strawberry guava, PW: pure water). The diagonal, dashed line represents the least squares regression line.

###
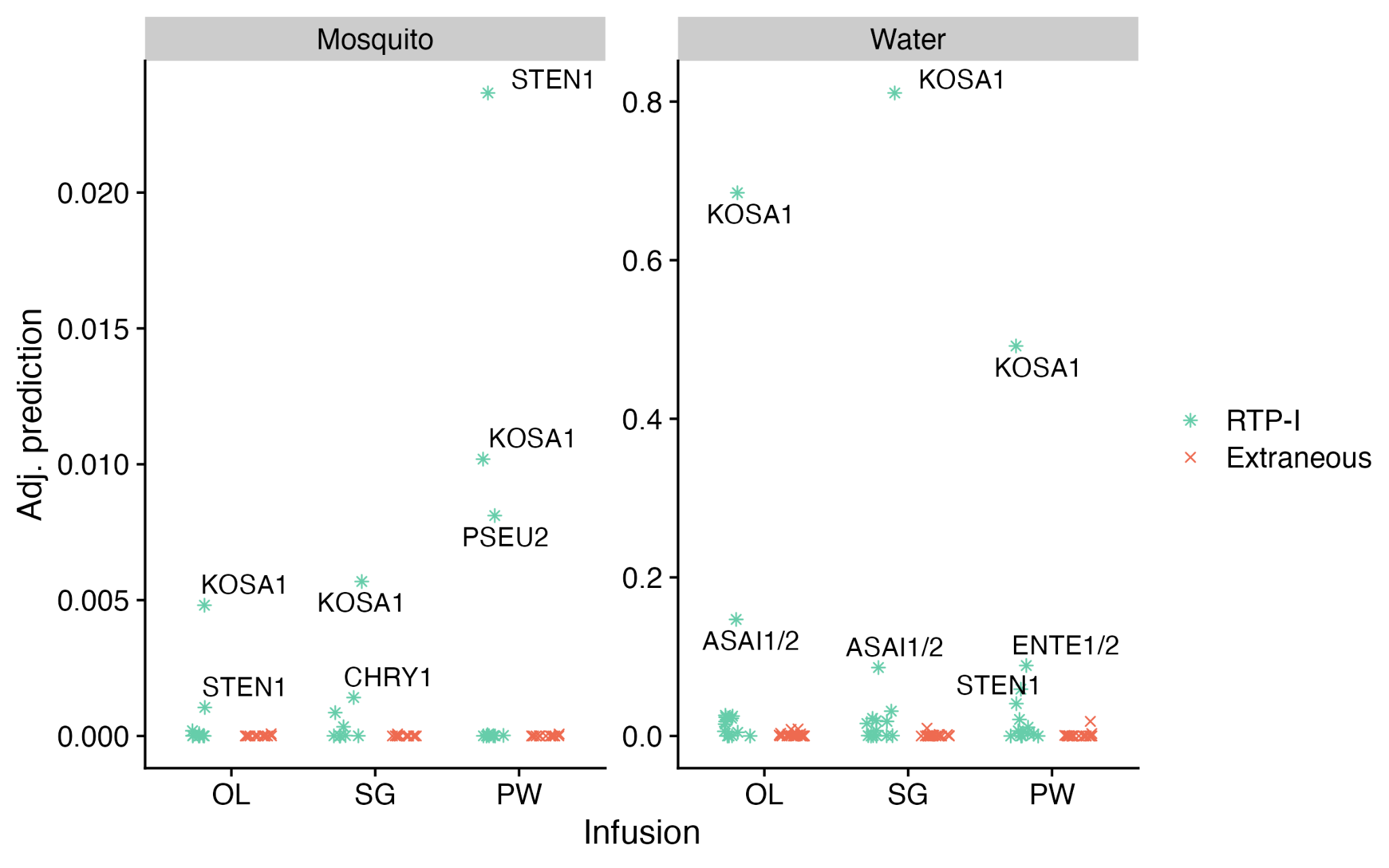


### Figure S6. Adjusted predicted probabilities of detecting a single sequencing read from an ASV per infusion type (OL: ‘ōhi‘a lehua, SG: strawberry guava, PW: pure water) based on GLMM results of the mosquito (left) or mesocosm water (right) samples. Points are shaped and colored by whether they were intentionally inoculated (“RTP-I”) or extraneous relative to the experiment. Higher values of the RTP-I taxa are labeled with their shorthand culture name.

###


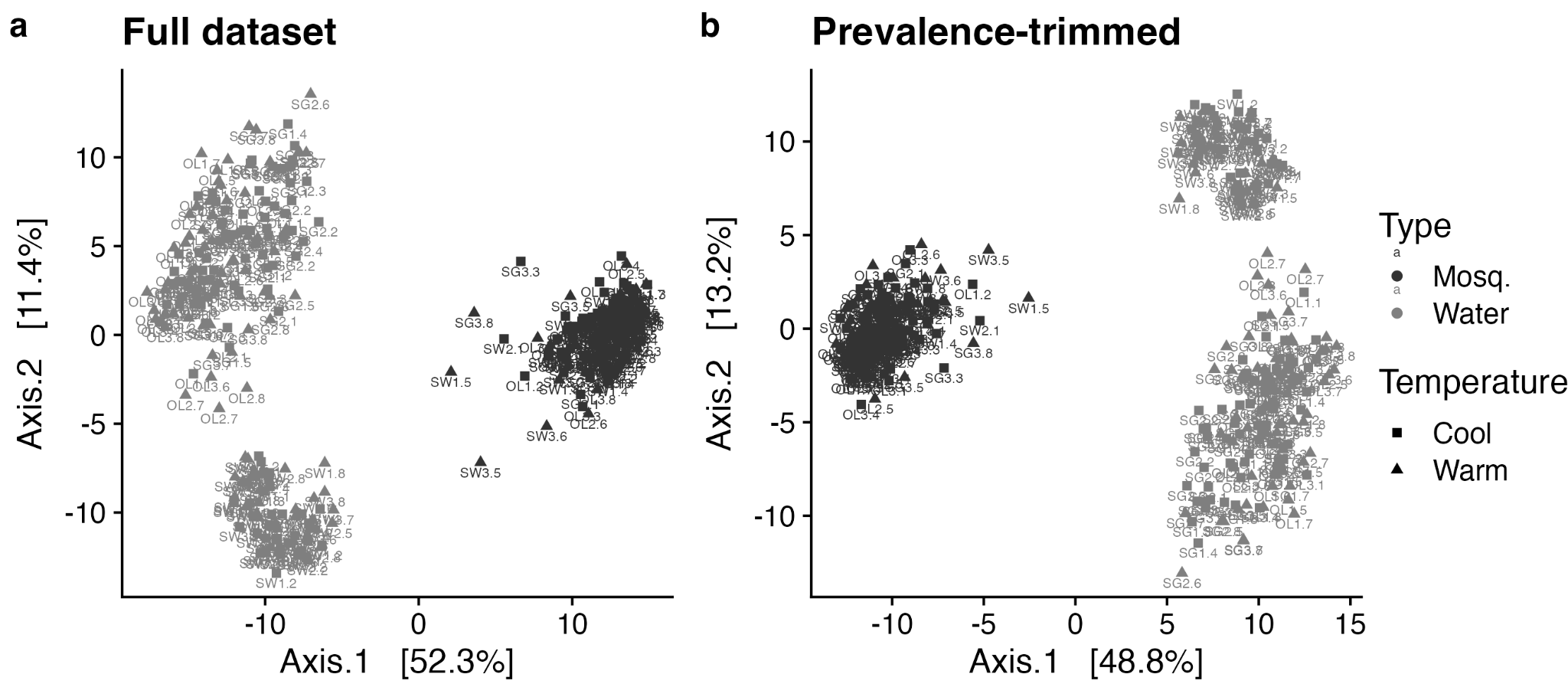


### Figure S7. Aitchison distances between samples plotted with the first two axes of a principal coordinate analysis (PCoA) for a the full dataset and b the dataset retaining taxa in >3.5% of samples from the mosquito (“Mosq.”) or mesocosm water (“Water”) sample types. Points are labeled by their mescosm ID, which begins with “OL” for ‘ōhi‘a lehua infusions, “SG” for strawberry guava and “SW” for pure water.

###
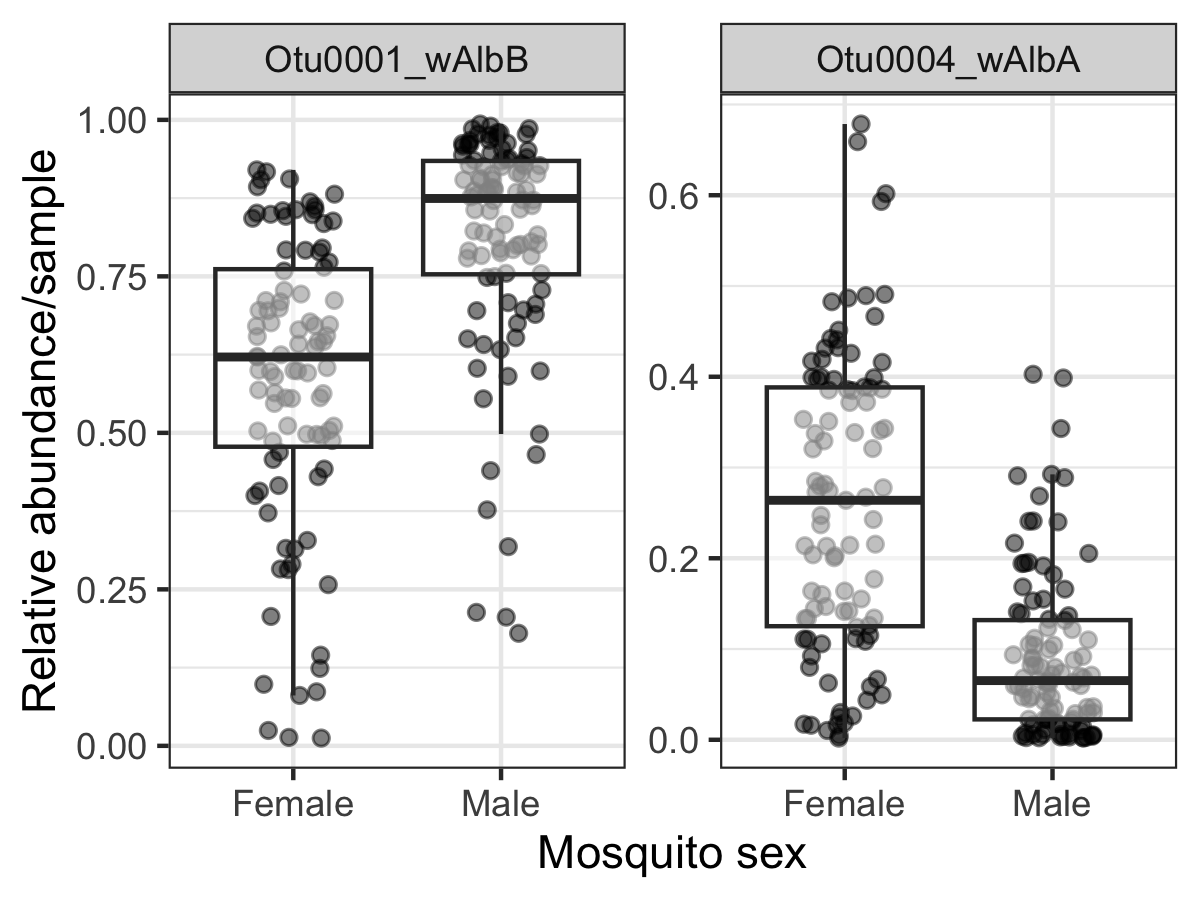


### Figure S8. Relative abundances per sample for *Wolbachia* strains wAlbB (left) and wAlbA (right) compared between mosquito sexes.

###

###

###
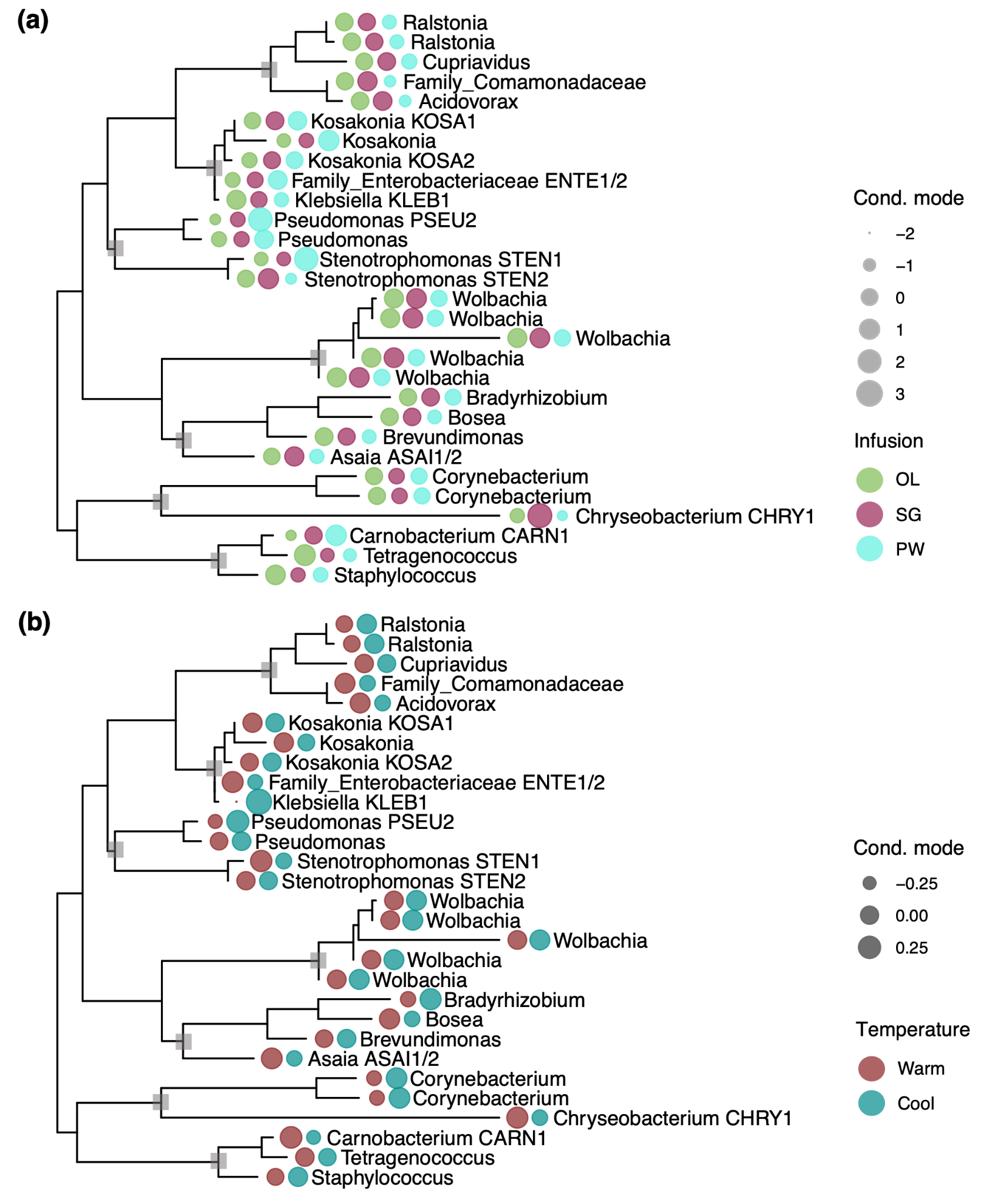


### Figure S9. Phylogenetic tree of mosquito-associated ASVs, labeled with their respective genus (or lowest available taxonomic group) and culture strain, where applicable. Colored circles at the branch tips scale to the size of the conditional mode of that ASV in their GLMM interactions with treatments of (a) infusion (OL: ‘ōhi‘a lehua, SG: strawberry guava, PW: pure water) and (b) temperature. Gray squares at branch nodes denote where taxa were grouped taxonomically to assess variance partitioned by clade.

###
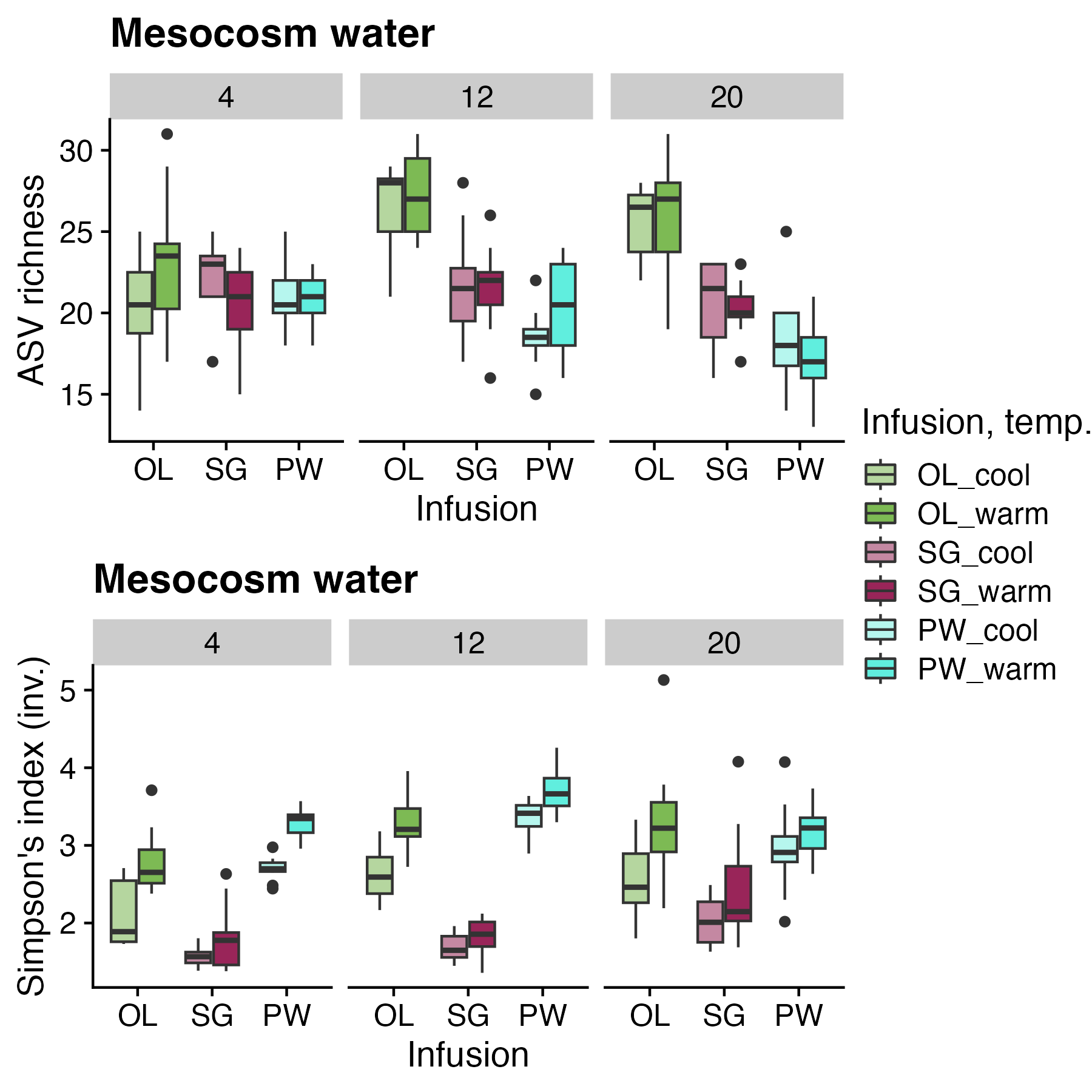


### Figure S10. Alpha diversity (top: ASV richness, bottom: Simpson’s index, inverted) in the mesocosm water through time (sampled on experimental days 4, 12, and 20) and across infusion (OL: ‘ōhi‘a lehua, SG: strawberry guava, PW: pure water) and temperature treatments.

### Table S3. Results from three statistical comparisons of microbiome community composition of the taxa with > 3.5% prevalence in mesocosm water samples across experimental variables. For the Generalized Linear Mixed Model (GLMM) results, the variance (± standard deviation) of the random effects’ conditional models is presented. For Aitchison distance and weighted UniFrac metrics, the *R*^2^ is given. Each of these results is followed by the associated *P* value and level of significance. “NA” indicates an inability to assess a variable due to aggregation of data by mesocosm identity. “^” indicates significant beta dispersion differences between groups, which may qualify the significance of the multivariate analysis.

| **Variable** | **GLMM** | **Aitchison distance** | **Weighted UniFrac** |
| --- | --- | --- | --- |
| Infusion | 8.14 (±2.85)  *P*<0.001*** | *R*^2^=0.65  *P*<0.001***(^) | *R*^2^=0.78  *P*<0.001*** |
| Temperature | 0.28 (±0.53)  *P*<0.001*** | *R*^2^=0.02  *P*<0.01**(^) | *R*^2^=0.06  *P*<0.001*** |
| Dispersal | 0.07 (±0.27)  *P*<0.001*** | *R*^2^=0.01  *P*=0.053 | R^2^=0.002  *P*=0.33 |
| Time period | 2.50 (±1.58)  *P*<0.001*** | NA | NA |

###
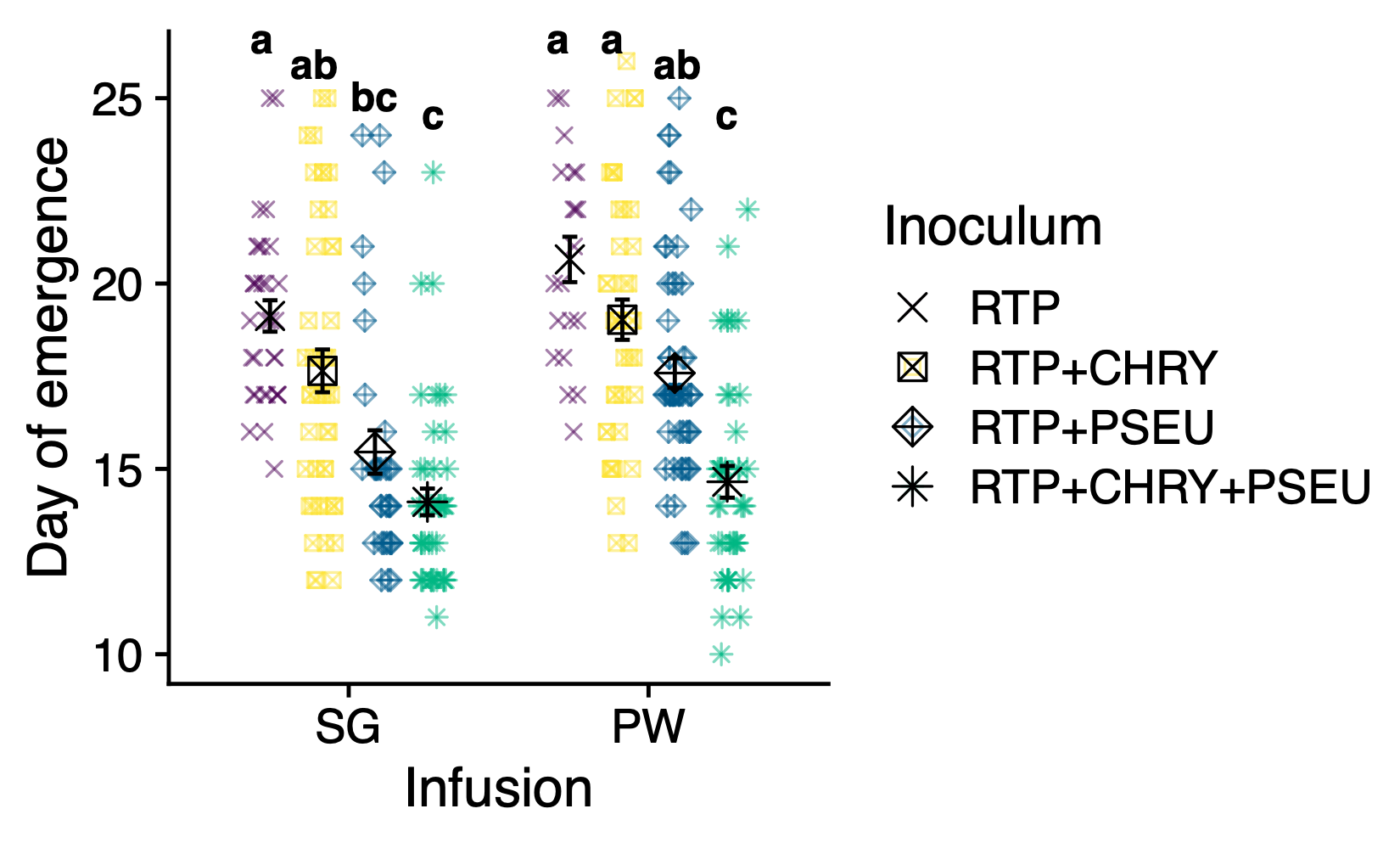


### Figure S11. Number of days for adult *Ae. albopictus* to emerge compared between inocula and infusions (SG: strawberry guava, PW: pure water) for Experiment II. For inocula, RTP indicates the base RTP-II inoculation, +CHRY indicates the addition of CHRY1, and +PSEU indicates the addition of PSEU2. Central symbols represent the mean, with error bars representing standard error. Different letters above the symbols indicate a significant difference in Tukey test *post hoc* comparisons based on package *emmeans* (*p*<0.05 [[10]](https://paperpile.com/c/cOJawy/233oE)).
